# Population dynamics and transcriptomic responses of *Pseudomonas aeruginosa* in a complex laboratory microbial community

**DOI:** 10.1038/s41522-018-0076-z

**Published:** 2019-01-14

**Authors:** Yingying Cheng, Joey Kuok Hoong Yam, Zhao Cai, Yichen Ding, Lian-Hui Zhang, Yinyue Deng, Liang Yang

**Affiliations:** 1 Singapore Centre for Environmental Life Sciences Engineering (SCELSE), Nanyang Technological University, Singapore, Singapore 637551; 2 Guangdong Province Key Laboratory of Microbial Signals and Disease Control, Integrative Microbiology Research Centre, South China Agricultural University, Guangzhou, China 510642; 3 Guangdong Innovative and Entrepreneurial Research Team of Sociomicrobiology Basic Science and Frontier Technology, South China Agricultural University, Guangzhou, China 510642; 4 School of Biological Sciences, Nanyang Technological University, Singapore, Singapore 637551; 5 School of Medicine, Southern University of Science and Technology, Shenzhen, China 518055

## Abstract

*Pseudomonas aeruginosa* tends to be among the dominant species in multi-species bacterial consortia in diverse environments. To understand *P. aeruginosa’s* physiology and interactions with co-existing bacterial species in different conditions, we established physiologically reproducible 18 species communities, and found that *P. aeruginosa* dominated in mixed-species biofilm communities but not in planktonic communities. *P. aeruginosa’s* H1 type VI secretion system was highly induced in mixed-species biofilm consortia, compared with its monospecies biofilm, which was further demonstrated to play a key role in *P. aeruginosa*'s enhanced fitness over other bacterial species. In addition, the type IV pili and Psl exopolysaccharide were required for *P. aeruginosa* to compete with other bacterial species in the biofilm community. Our study showed that the physiology of *P. aeruginosa* is strongly affected by interspecies interactions, and both biofilm determinants and type VI secretion system contribute to higher *P. aeruginosa*'s fitness over other species in complex biofilm communities.

## Microbial ecology: transcriptome determinants for biofilm dominance

*Pseudomonas aeruginosa* bacteria are highly adaptable and dominate different environments—notably biofilms—yet the biological and ecological factors that support this dominance are still poorly understood. Here, Yingying Cheng and Liang Yang at the Nanyang Technological University, Singapore, used multiplexed gene expression analysis of mixed-species microbial communities to monitor transcriptional changes in *P. aeruginosa*, and reveal the upregulation of the H1 type VI secretion system and the expression of the type IV pili and Psl exopolysaccharide as central events in establishing the dominance of *P. aeruginosa* over other bacterial species growing in biofilms. This work provides new insights into the factors that enhance the fitness of *P. aeruginosa* in multi-species communities, and exemplifies how transcriptome analyses can uncover the physiological properties that enable *P. aeruginosa* to overcome other microbial species and thrive in complex biofilms.

## Introduction

Biofilms can protect bacteria from hostile environments and also disperse bacteria to colonise in new niches.^1,2^ *Pseudomonas aeruginosa* is a model organism for studying biofilm formation in Gram-negative bacteria.^1,3^ The biofilm mode affords *P. aeruginosa* protection and renders it more difficult to control.^4,5^ Type IV pili (T4P), flagella, iron, extracellular DNA and Pel/Psl exopolysaccharides (EPS) are well known factors that contribute to the development of *P. aeruginosa* monospecies biofilms.^6–8^ The adaptability of *P. aeruginosa* to various environments, such as water, soil, sewage and plants, suggests its strong competitiveness for ecological niches.^6,9,10^ For example, *P. aeruginosa* is a dominant cultivable endophytic bacterium associated with *Pennisetum glaucum* (millet).^11^

The broad-spectrum adaptability of *P. aeruginosa* also hints at its strong competitiveness against various other bacterial species when in mixed-species microbial communities. Several studies have indicated that *P. aeruginosa* is able to gain fitness over competing species during its survival in either two-species or three-species co-cultures.^12–14^ For example, *P. aeruginosa* reduces the viability of *Staphylococcus aureus* and lyses *S. aureus* cells for iron repletion in planktonic co-cultures.^15,16^ In biofilms, *P. aeruginosa* inhibits *Staphylococcus epidermidis* growth and reduces the initial adhesion of *Agrobacterium tumefaciens* through the secretion of Psl and Pel exopolysaccharides and small diffusible molecules.^17–19^ *P. aeruginosa* decreases the swarming motility of *P.* *putida* and therefore inhibits its biofilm formation by producing 2-heptyl-3-hydroxy-4-quinolone.^20^ Quinolones secreted by *P. aeruginosa* can also reduce biofilm maturation for a broad range of bacteria.^21^ These studies demonstrate the competitiveness of *P. aeruginosa* in mixed-species interactions, from metabolic versatility to a strong biofilm formation capacity. Documented strategies in which *P. aeruginosa* exerts competition involve type VI secretion system (T6SS), generation of antibiotics, iron chelators, pyoverdine, rhamnolipid, pyocyanin, extracellular polysaccharide, fatty acid *cis*-2-decenoic acid, proteinase and other quorum-sensing system-regulated virulence factors.^22–27^

*P. aeruginosa* co-exists with many different microbial species in natural settings. However, the main competitive advantage of *P. aeruginosa* in gaining fitness over other species within complex microbial communities remains unclear. Previous studies indicated that interspecies interactions in mixed-species microbial communities are complicated and involve both cooperation and competition.^24,28^ Therefore, monitoring the population dynamics of *P. aeruginosa* in complex microbial communities is important for elucidating how this interaction network is established, which may provide insights into its ecological role in complex mix-species biofilms.

Until recently, there has been a lack of robust tools to monitor the population dynamics in complex microbial communities. The high-throughput digital NanoString nCounter® system has originally been developed to profile the eukaryotic multiplexed gene expression with flexibility and sensitivity.^29,30^ However, this technology has never been reported for its utilisation on monitoring population dynamics in a complex microbial community. Here we modified the probes to achieve a new application in investigation microbial ecology and evaluated this technology in monitoring population dynamics in a complex laboratory mixed-species microbial community with pairs of tailored probes directly hybridise to the 16 S rRNA of each species instead of mRNA without any enzymatic reaction or DNA synthesis.^29^ We further investigated the transcriptome of *P. aeruginosa* competition in this mixed-species community in both planktonic and biofilm modes of growth.

## Results

### Establishment of the complex microbial community

Instead of proliferating as a single-species culture, *P. aeruginosa* often grows as commensal species within mixed-species microbial communities containing many other bacterial species in natural environments as well as infection sites.^31,32^ We presumed that bacteria within the same region of colonisation would have the same chance to co-exist. To obtain a better understanding of *P. aeruginosa* physiology in complex microbial communities and evaluate the NanoString nCounter® system, we selected bacterial species that potentially co-exist with *P. aeruginosa* in different environments and can be distinguished from other species by NanoString nCounter® probes. Hence, 18 human pathogens/environmental bacteria (Supplementary Table 1) were established as laboratory planktonic and biofilm communities as some of these species usually share the same niche in humans or in natural environments. Among these species, *Acinetobacter baumannii*, *Burkholderia cenocepacia*, *Klebsiella pneumoniae* and *S. aureus* can co-infect lungs with *P. aeruginosa*^33–36^; *Citrobacter amalonaticus*, *E. coli* and *Streptococcus gallolyticus* can cause intra-abdominal infections; *Chromobacterium violaceum*, *S. aureus* and *Stenotrophomonas maltophilia* can infect the skin or open wounds; *Listeria monocytogenes* can colonise a host’s gastrointestinal tract; *P. putida* has been isolated from infected urinary tract and infected skin; *Bacillus subtilis*, *Elizabethkingia meningoseptica*, *P. fluorescens* and *Shewanella oneidensis* are widely distributed in natural environments; and *P. syringae* and *Xanthomonas campestris* have been associated with plants.^37^ The abovementioned settings all involve the potential co-existence of *P. aeruginosa*. Both known and undiscovered natural environments are so complex and unexpected. This laboratory community may assist in understanding *P. aeruginosa’s* interactions with complex microbial communities. In addition to investigating the ecological behaviour and physiological response of *P. aeruginosa* in our model system, we also seek insights into how future studies on complex microbial communities can be shaped.

We used 10 times diluted tryptic soy broth (10% TSB) as the growth medium, which supports the growth of all the selected 18 bacterial species at 25 °C (room temperature) (Supplementary Figure 1). Although *A. baumannii*, *C. amalonaticus*, *C. violaceum*, *E. meningoseptica*, *E. coli* and *K. pneumoniae* grew relatively faster than other species, and *L. monocytogenes* and *S. gallolyticus* were unable to grow to a high cell density in 10% TSB, the growth curves indicated that 10% TSB was suitable for establishing the mixed-species microbial community, with *P. aeruginosa* growth comparable to the majority of the species (Supplementary Figure 1).

In addition to the growth rate, the biofilm formation capacity of these bacterial species was also verified in monospecies culture. After 24 h of static incubation, *A.* *baumannii* and *K. pneumoniae* had the highest capacity to form biofilms (Supplementary Figure 2). Among the other 16 species, *B. cenocepacia*, *P. aeruginosa* and *S. maltophilia* have similar biofilm formation capabilities (Supplementary Figure 2). However, *B. subtilis*, *C. violaceum*, *E. meningoseptica*, *P. putida*, *P. syringae* and *S. gallolyticus* formed less biofilms in 10% TSB at 25°C (Supplementary Figure 2). In summary, the biofilm formation capacities of the 18 species differ, with no linear relationship with growth rates. We therefore established the planktonic and biofilm microbial communities by adjusting each species to an OD_600_ value of 0.01 in 10% TSB medium as inocula.

### Physiological reproducibility of the mixed-species microbial communities

**Fig. 1:**
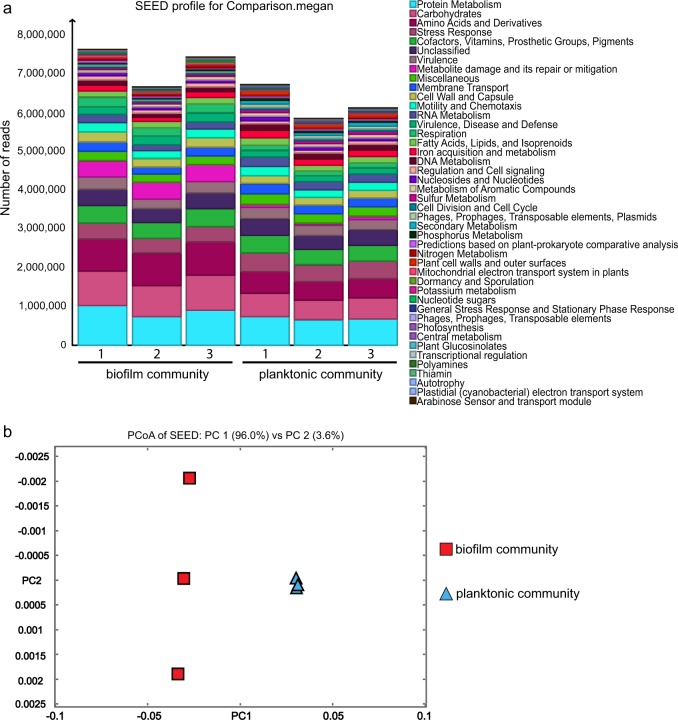
Metatranscriptomics comparison between mixed-species planktonic community and biofilm community modes. **a** Functional classification by SEED. The comparison was based on read counts with a minimum quality score cutoff of 50 and a top percentage of 25. Different colours corresponding to different functions are ranked in the right column. **b** Clustering by PCoA based on SEED classification using Bray–Curtis dissimilarity

Before investigating the population dynamics and physiology of *P. aeruginosa* in the mixed-species communities, we employed RNA sequencing-based metatranscriptomics to examine the physiological reproducibility of both the planktonic and biofilm communities. The sequencing reads (7.8–9.2 million per sample) were assigned to 44 classified functional roles (SEED; 51.4 million reads) with unclassified reads in MEGAN6. Similar profiles of functional classifications of genes assigned to each community model by their functional properties were observed among three biological replicates (Fig. 1a), indicating that physiology is highly reproducible in our laboratory model for both planktonic and biofilm communities (Fig. 1a). The proportion of each small bar with different colours represents the ratio of different functional genes in communities. For example, expression level of 'metabolite damage and its repair or mitigation' genes is higher in biofilm communities than in planktonic communities. In contrast, expression levels of genes in 'iron acquisition and metabolism', 'cell division and cell cycle', 'secondary metabolism' are higher in planktonic communities than in biofilm communities (Fig. 1a). In addition, clear separation between the two clusters on the principal coordinates analysis (PCoA) plot confirmed that there is a significant difference between the metatranscriptome profiles of the planktonic community and the biofilm community. The close proximity among replicates of each community indicates the similarity in metatranscriptome profiles of the replicates (Fig. 1b). The relative distanced location among replicates of biofilm community is owing to the small scale chosen to display all points clearly along PC2. Above result suggests a good reproducibility in our model of mixed-species cultures which can serve as a stable system for functional analysis carried out in this study.

### Evaluation of NanoString nCounter® 16S rRNA array for microbial population assay

Although the 16 S rRNA gene amplicon sequencing approach is widely used to study the microbial population diversity, it has the drawback of PCR bias. A NanoString nCounter® 16 S rRNA array was developed to detect unique signals from complex hybridised samples at a single-molecule level by utilising special probes captures target 16 S rRNA sequence and omitting the PCR amplification step^38^ (Supplementary Table 1). To check the feasibility of the designed nCounter® 16 S rRNA array for detecting the 16 S rRNA from different bacterial species in the total RNA mixture, we mixed the total RNA extracted from 14 individual bacterial species at different ratios in three different training groups (Table 1). These were subjected to nCounter® 16 S rRNA array analysis, with the total RNA from the planktonic and biofilm communities together in one batch. The percentage of each species in 16 S rRNA reads according to nCounter® 16 S rRNA array analysis was compared with its ‘actual’ RNA percentage from the training groups to calculate the normalisation factors for each of the 14 species (Table 1). To verify this new technology, the *P. aeruginosa* probe was tested against the background of other genera in the mixed-species community, with the other three *Pseudomonas* species excluded. One Gram-positive probe was tested against the background of other genera with *L. monocytogenes’s* absence. The nCounter® 16 S rRNA array yielded more reads to some species than accurate 16 S rRNA readings, whereas the converse occurred for other species (Table 1). This trend highlighted the need to analyse bacterial population dynamics by multiplying the normalisation factors (the variation fold) (Table 1), when nCounter® 16 S rRNA array is employed to reflect the true percentage of each bacterial species in a mixed-species community.

**Table 1:** nCounter^®^ 16S rRNA array feasibility detection

	Reads percentage (%)			RNA percentage (%)			Fold (normalisation factor)
	I	II	III	I	II	III	
*A. baumannii*	3.13	6.52	1.09	1.95	3.71	1.92	1.31 ± 0.65
*B. subtilis*	0.18	0.07	0.06	0.11	0.10	0.10	1.00 ± 0.61
*B. cenocepacia*	3.88	0.54	1.01	1.93	1.76	9.55	0.81 ± 1.04
*C. violaceum*	16.94	3.43	3.05	21.48	9.77	10.60	0.48 ± 0.27
*C. amalonaticus*	7.56	6.36	0.10	11.45	20.84	-	0.48 ± 0.25
*E. coli*	2.10	2.50	0.97	1.88	6.83	3.71	0.58 ± 0.47
*E. anophelis*	0.87	0.00	0.00	0.41	-	-	2.10 ± -
*K. pneumoniae*	6.90	0.29	0.11	40.48	-	-	0.17 ± -
*L. monocytogenes*	0.01	0.02	0.03	-	-	-	-
*P. aeruginosa*	23.28	47.95	72.63	6.53	29.69	57.95	2.14 ± 1.24
*P. fluorescens*	0.44	0.92	1.46	-	-	-	-
*P. putida*	2.22	4.98	6.13	-	-	-	-
*P. syringae*	0.01	0.01	0.01	-	-	-	-
*S. oneidensis*	0.28	0.35	0.24	1.24	2.25	2.44	0.16 ± 0.06
*S. aureus*	13.24	5.18	2.09	3.73	3.40	1.84	2.07 ± 1.30
*S. maltophilia*	0.47	0.01	0.00	0.59	-	-	0.79 ± -
*S. gallolyticus*	2.67	10.34	0.91	0.58	4.25	0.58	2.86 ± 1.54
*X. campestris*	15.82	10.52	10.10	7.65	17.40	11.32	1.19 ± 0.78

Three different training groups were named 'I', 'II' and 'III' in this table. Numbers indicate the ratio of one species in the mixed group with unit %. 'Reads percentage' is nCounter^®^ 16 S rRNA array analysis result; 'RNA percentage' shows proportion of single species total RNA in mixture. 'Fold' calculated from average values of ratio in Reads percentage to it in RNA percentage. Dash in RNA percentage indicates no RNA was mixed into the random mixture sample, i.e., some average fold values and standard deviations could not been calculated, also labelled with a dash

### *P. aeruginosa* is the dominant species in the mixed-species biofilm community

**Fig. 2:**
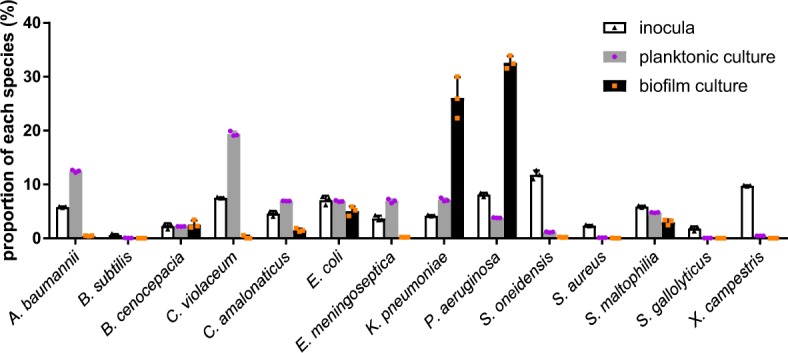
Normalised population dynamics of each species in the mixed-species microbial communities. The inocula is the mixture of all species mixed at equal OD_600_ amounts at the starting point for both planktonic and biofilm incubation. Grey bars indicate the proportion of each species in the 24-hour planktonic microbial community. The black bars show the population dynamics of each species in the 5-day biofilm microbial community. Dots show three biological replicates for standard deviation

To maintain the reproducibility of this laboratory model, we used the same mixed-species community. Each individual species culture was normalised to an OD_600_ reading of 0.01 in the mixture and cultivated for 1 d to establish the planktonic community, and 5 d to establish the biofilm community. Total RNA extracted from these communities were analysed by nCounter® 16 S rRNA array. In the 1-day-old planktonic community, the relative abundance of *A. baumannii*, *C. violaceum* and *E. meningoseptica* increased by twofold or more compared with their inocula, whereas the relative abundance of *B. subtilis*, *P. aeruginosa*, *S. oneidensis*, *S. aureus*, *S. gallolyticus* and *X. campestris* decreased to half or less compared with their inocula (Fig. 2 and Supplementary Table 2). Interestingly, the microbial population dynamics are quite different in the 5-day-old biofilm community compared with the planktonic community; the relative abundance of *K. pneumoniae* and *P. aeruginosa* increased to more than fourfold their initial inputs, whereas most of the other species decreased significantly (Fig. 2 and Supplementary Table 2). *P. aeruginosa* 16 S rRNA constituted ~32.62% of the total 16 S rRNA content, whereas *K. pneumoniae* constituted ~26.08% of that in 5-day-old biofilms (Fig. 2, and Supplementary Table 2) after normalisation factor adjustment (Table 1). The proportions of all bacterial species (including the four non-normalised ones) are summarised in Supplementary Figure 3.

**Fig. 3:**
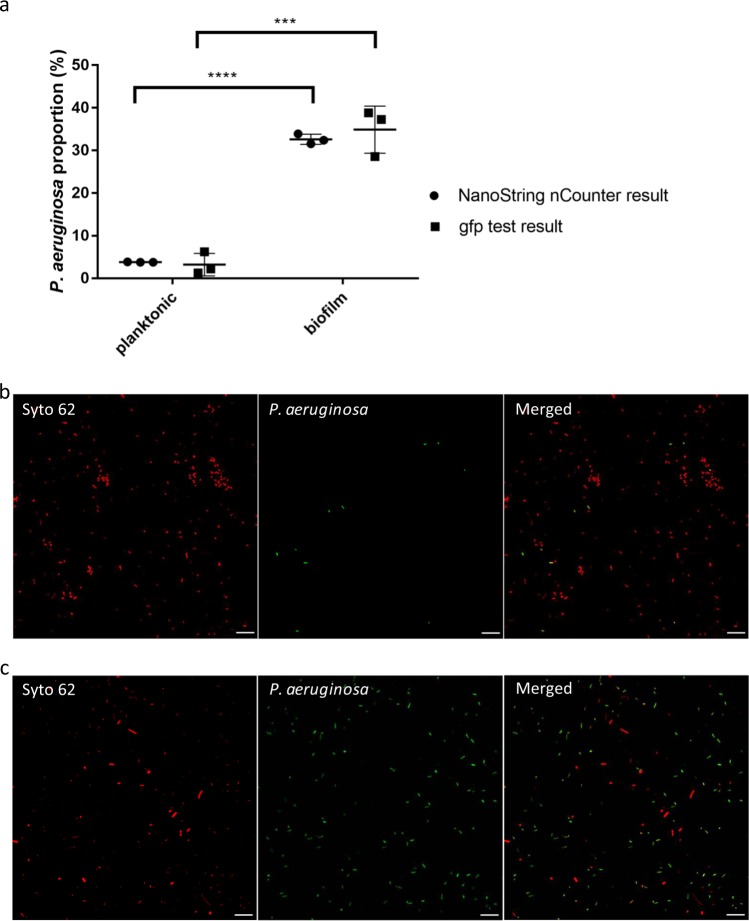
Proportions of *P. aeruginosa* in the mixed-species microbial communities. **a** Circular dots indicate three biological replicates of *P. aeruginosa* normalised proportions in both the planktonic and biofilm communities as measured by NanoString nCounter® 16 S rRNA array; the square dots show three biological replicates of *P. aeruginosa* proportions in mixed-species microbial communities as calculated by fluorescence-based proportion test. The lines show the average values and standard deviation. Comparison was performed by *t* test with *p* < 0.05. **b** Confocal images of the resuspended 24 h-incubated mixed-species planktonic microbial community containing GFP-tagged *P. aeruginosa*. **c** Confocal images of the resuspended 5-day mixed-species biofilm community containing GFP-tagged *P. aeruginosa*. Green cells show the GFP-tagged *P. aeruginosa*, red cells show all of other cells stained with Syto62. Scale bar represents 10 µm

To validate the observed microbial population dynamics, we used GFP-tagged *P. aeruginosa* to establish the 1-day-old planktonic community and the 5-day-old biofilm community as described above. The abundance of *P. aeruginosa* was detected and calculated using the fluorescence-proportion standard curve (Supplementary Figure 4). The fluorescence-proportion standard curve is preferred over the colony-forming unit counting approach owing to the difference in the growth rates and the colony sizes of these 18 bacterial species on plates, which contributes to the accuracy of dynamics population counting. Resuspended cells from both planktonic and biofilm communities were used for fluorescence-based proportion tests and next examined using confocal microscopy imaging (Fig. 3b, c). The results of the biological fluorescence-proportion standard curve-based experiment correlated well with the results of the normalised nCounter® 16 S rRNA array (Fig. 3a). The confocal images analysis further verified the proportion of *P. aeruginosa* cells in the mixed-species biofilm community (Fig. 3c) is much higher than in the planktonic community (Fig. 3b). Together with the results in Fig. 2, we have demonstrated that *P. aeruginosa* is the dominant species in this microbial biofilm community.

### Both the H1 type VI secretion system and biofilm formation determinants contributed to the fitness of *P. aeruginosa* in the biofilm community

**Fig. 4:**
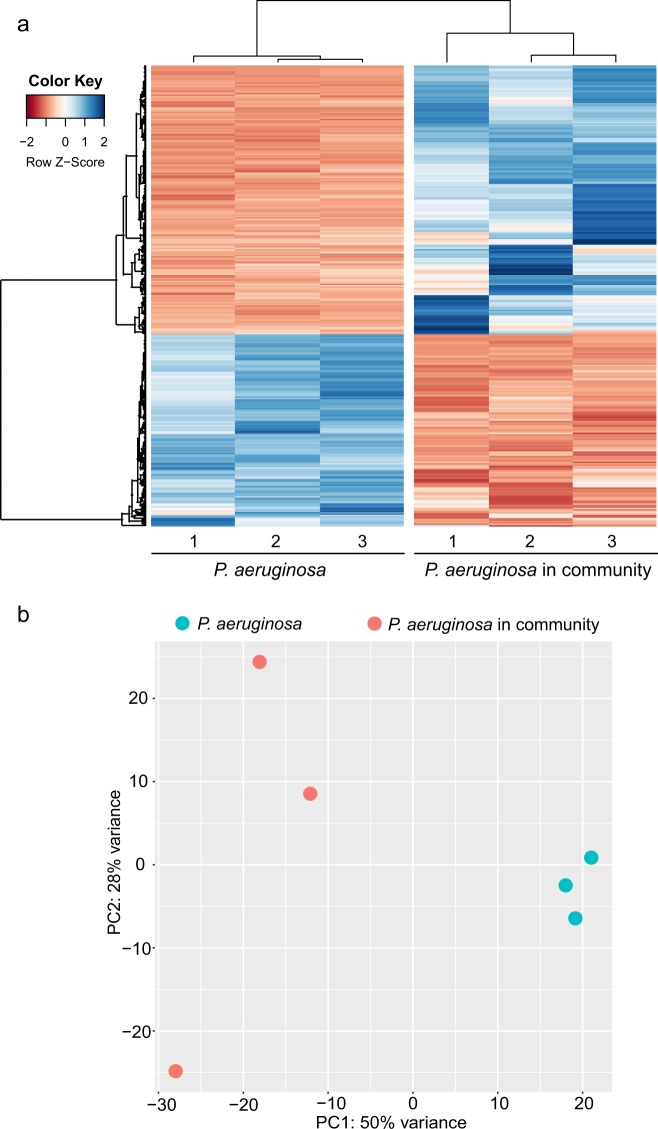
Transcriptomic comparison of *P. aeruginosa* between monospecies biofilm cells and the mixed-species biofilm community. **a** A heat map representing *P. aeruginosa* genes expression level up- or downregulated more than twofold in the mixed-species biofilm compared with the monospecies biofilm. The *Z s*core shows the standardisation of each gene expression level between the two different groups. Detailed information on this regulation is listed in Supplementary Table 3. **b** A principal component analysis (PCA) shows that the expression profiles of *P. aeruginosa* between the monospecies biofilm and in the mixed-species biofilm are different. Each dot indicates one biological replicate with different colours representing the sample source

As *P. aeruginosa* constituted up to 32.62% of the biofilm community in contrast to only 3.81% in the planktonic community (Supplementary Table 2), we hypothesised that the important biofilm determinants are likely to contribute to the dominating competence of *P. aeruginosa* over other species in the biofilm community. To test this hypothesis, we performed a transcriptomics analysis comparing the gene expressions in monospecies *P. aeruginosa* biofilms with that of the mixed-species biofilm community. Differentially expressed genes of *P. aeruginosa* in mixed-species biofilm and monospecies biofilm are illustrated as a heat map (Fig. 4a). Further clustering of the transcriptome by principle component analysis revealed that the *P. aeruginosa* maintained a distinct physiology in monospecific and mixed-species biofilms (Fig. 4b). Based on the negative binomial test with an adjusted *P* value cutoff of 0.05 and a log2-fold change cutoff of 1,239 genes were upregulated and 171 genes were downregulated in *P. aeruginosa* cells in the mixed-species biofilm compared to those in monospecific biofilms (Supplementary Table 3).

**Fig. 5:**
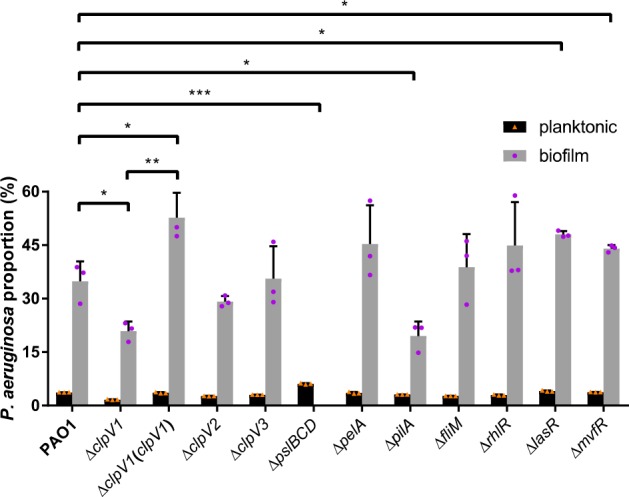
*P. aeruginosa* population dynamics in the mixed-species biofilm community affected by H1-T6SS and biofilm formation determinants. Each column shows the population dynamics of GFP-tagged *P. aeruginosa* parental strain PAO1 and the knockout mutants in the mixed-species microbial planktonic (black) and biofilm (grey) communities at different heights with the standard deviation of three biological replicates (dots). The asterisk indicates statistical significance by *t* test with p < 0.05

Among the significantly upregulated genes of *P. aeruginosa* in the mixed-species biofilm, at least 36 belonged to the T6SS (Table 2), which accounted for 15% of all the upregulated genes. Most of these belong to the Hcp secretion island I (H1) T6SS, whereas some are located in the Hcp secretion island II (H2) T6SS. However, there were only a few genes that belong to H3-T6SS. The ClpV protein is the energy source of T6SS^39^; the expressions of *clpV1*, *clpV2* and *clpV3* belonging to three different sub-type T6SS in *P. aeruginosa* were all upregulated in the mixed-species biofilm (Table 2). To validate the transcriptomic analysis results, the Δ*clpV1*, Δ*clpV2* and Δ*clpV3* mutants of *P. aeruginosa* were constructed with a GFP tag and thereafter formed biofilms with the other 17 species. Interestingly, only the Δ*clpV1* mutant was greatly impaired in terms of fitness to dominate in the mixed-species biofilms; when ClpV1 was complemented into this mutant with multiple copies, the complementation strain showed higher fitness to dominate in the mixed-species biofilm community than both wild-type PAO1 and the deletion mutant (Fig. 5). This finding suggests that the H1-T6SS has a significant effect on fitness gains for *P. aeruginosa*, enabling it to outcompete other species in the mixed biofilm community (Fig. 5). In addition, confocal images confirmed that parental strain PAO1 gains higher fitness over the other bacterial species than its isogenic H1-T6SS defect mutant Δ*clpV1* in the 18 species biofilm communities (Supplementary Figure 5c and 5d). H1-T6SS contains seven effectors to be secreted into its competitor, which are termed Tse1-7.^40,41^ Both Tse1 and Tse3, which can lyse the target cells by degrading their peptidoglycan,^26^ were upregulated in the complex microbial biofilm community (Table 2). Furthermore, the expression of the gene encoding the H1-T6SS delivery-dependent proteins VgrG1 was also increased (Table 2).

**Table 2:** Some of the *P. aeruginosa* regulated genes in mixed-species biofilm community

Locus tag	Gene name	Fold change	*p* value	Product	Pathway
PA0070	*tagQ1*	2.66	1.20E-09	TagQ1	H1-T6SS
PA0074	*ppkA*	2.06	1.77E-04	Serine/threonine protein kinase PpkA	H1-T6SS
PA0077	*icmF1*	2.43	2.68E-07	IcmF1	H1-T6SS
PA0079	*tssK1*	2.06	4.11E-04	TssK1	H1-T6SS
PA0082	*tssA1*	2.27	5.28E-04	TssA1	H1-T6SS
PA0083	*tssB1*	5.34	1.81E-23	TssB1	H1-T6SS
PA0084	*tssC1*	5.24	1.51E-31	TssC1	H1-T6SS
PA0085	*hcp1*	4.82	1.20E-52	Hcp1	H1-T6SS
PA0086	*tagJ1*	2.43	4.84E-04	TagJ1	H1-T6SS
PA0088	*tssF1*	2.85	6.79E-07	TssF1	H1-T6SS
PA0089	*tssG1*	2.62	8.73E-04	TssG1	H1-T6SS
PA0090	*clpV1*	4.96	1.52E-21	ClpV1	H1-T6SS
PA0091	*vgrG1*	3.53	1.57E-12	VgrG1	H1-T6SS
PA0092	*tsi6*	2.4	8.41E-04	Tsi6	H1-T6SS
PA0097	PA0097	2.48	7.21E-05	Hypothetical protein	
PA0099	PA0099	3.61	2.72E-15	Type VI effector protein	
PA0100	PA0100	2.86	8.24E-15	Hypothetical protein	
PA0260	*tle3*	2.62	2.67E-17	Tle3	H2-T6SS
PA0261	PA0261	2.34	2.26E-05	Hypothetical protein	
PA0263	*hcpC*	4.23	1.31E-18	Secreted protein Hcp	
PA1509	*tplEi*	2.12	1.83E-07	Immunity protein TplEi	
PA1510	*tplE*	2.8	1.54E-12	Type 6 PGAP1-like effector, TplE	H2-T6SS
PA1512	*hcpA*	3.52	1.95E-14	Secreted protein Hcp	
PA1657	*tssB2*	3.06	7.18E-18	TssB2	H2-T6SS
PA1658	*tssC2*	2.65	1.06E-16	TssC2	H2-T6SS
PA1659	*tssE2*	2.18	8.21E-05	TssE2	H2-T6SS
PA1661	*tssH2*	2.35	1.57E-06	TssH2	H2-T6SS
PA1662	*clpV2*	2.36	1.79E-10	clpV2	H2-T6SS
PA1663	*sfa2*	2.61	5.56E-08	Sfa2	H2-T6SS
PA1665	*fha2*	2.74	3.11E-09	Fha2	H2-T6SS
PA1666	*lip2*	3.3	2.74E-11	Lip2	H2-T6SS
PA1667	*tssJ2*	2.8	5.26E-08	TssJ2	H2-T6SS
PA1668	*dotU2*	2.27	1.18E-05	DotU2	H2-T6SS
PA1669	*icmF2*	3.21	1.74E-14	IcmF2	H2-T6SS
PA1844	*tse1*	2.86	1.62E-03	Tse1	H1-T6SS
PA1845	*tsi1*	2.81	4.93E-04	Tsi1	H1-T6SS
PA2365	*tssB3*	3.78	6.97E-22	TssB3	H3-T6SS
PA2366	*tssC3*	3.75	1.81E-37	TssC3	H3-T6SS
PA2367	*hcp3*	3.08	1.35E-18	Hcp3	H3-T6SS
PA2369	*tssG3*	2	1.03E-04	TssG3	H3-T6SS
PA2371	*clpV3*	2.11	1.07E-08	ClpV3	H3-T6SS
PA3294	*vgrG4a*	2.17	1.02E-03	VgrG4a	H2-T6SS
PA3484	*tse3*	2.54	6.03E-04	Tse3	H1-T6SS
PA3479	*rhlA*	−2.35	4.99E-07	Rhamnosyltransferase chain A	Quorum sensing
PA3478	*rhlB*	−2.17	8.81E-07	Rhamnosyltransferase chain B	Quorum sensing
PA1871	*lasA*	−2.42	1.64E-08	LasA protease precursor	Quorum sensing
PA3724	*lasB*	−2.72	3.03E-12	Elastase LasB	Quorum sensing
PA1432	*lasI*	2.43	1.13E-14	Autoinducer synthesis protein LasI	Quorum sensing
PA2587	*pqsH*	−2.09	3.42E-05	Probable FAD-dependent monooxygenase	Quorum sensing
PA1078	*flgC*	2.08	1.14E-05	Flagellar basal-body rod protein FlgC	Flagella assembly
PA1079	*flgD*	2.26	2.60E-10	Flagellar basal-body rod modification protein FlgD	Flagella assembly
PA3059	*pelF*	2.44	2.40E-03	PelF	Extracellular polysaccharide biosynthesis
PA3058	*pelG*	2.82	1.84E-03	PelG	Extracellular polysaccharide biosynthesis

In addition to T6SS, the *P. aeruginosa pelF*, *pelG*, *flgC* and *flgD* genes, which are important for biofilm formation in monospecies cultures, were also upregulated in the mixed-species biofilms (Table 2). We next investigated the impact of the classic monospecies *P. aeruginosa* biofilm determinants, e.g., Pel and Psl EPS,^42–44^ T4P^8,45^ and flagella,^8,45^ on the fitness of *P. aeruginosa* in the mixed-species biofilms. The *P. aeruginosa* deletion mutants Δ*pslBCD*, Δ*pelA*, Δ*pilA* or Δ*fliM* were used to cultivate mixed-species biofilms with the other 17 bacterial species respectively. The Psl polysaccharide and T4P were required for *P. aeruginosa* to predominate in the mixed-species biofilm community (Fig. 5). Psl was particularly essential for bacterial colonisation in the mixed-species biofilms,^46^ and Δ*pslBCD* was completely outcompeted by other species in the biofilms (Fig. 5), even though its planktonic growth was similar to that of the wild-type PAO1 strain (Supplementary Figure 6).

### Quorum sensing is not required for the fitness of *P. aeruginosa* in the mixed-species biofilm community

Quorum-sensing systems play important roles in biofilm formation both in vitro and in vivo.^47–52^ *P. aeruginosa* is able to outcompete other microbial species by releasing quorum sensing-regulated virulence factors, such as rhamnolipid, elastase, exotoxins, pyocyanin and pyoverdine.^53–57^ However, the expression of the *P. aeruginosa* quorum sensing-related genes, such as *lasA*, *lasB*, *rhlA*, *rhlB* and *pqsH*, were downregulated in this biofilm community compared with the monospecies biofilm (Table 2), suggesting that quorum sensing is not important in contributing to the fitness of *P. aeruginosa* in the mixed-species community biofilm. Intriguingly, deleting the *lasR* or *mvfR* quorum-sensing genes in *P. aeruginosa* even increased this species’ fitness over other species in the mixed-species biofilms, compared to the PAO1 wild-type (Fig. 5). This could possibly be owing to *P. aeruginosa* H1-T6SS being repressed by LasR and MvfR.^58^

## Discussion

Our study reported on the establishment of physiologically reproducible laboratory planktonic and biofilm communities and their use in investigating how *P. aeruginosa* competes with multiple other bacterial species. We tracked the population dynamics and profiled the transcriptomes of the established communities and showed that *P. aeruginosa* maintains distinct transcriptomes in complex microbial communities compared to its monospecies cultures (Fig. 4). The H1-T6SS, Psl and T4P were found to be the key components for *P. aeruginosa* to gain fitness over other species in the mixed-species biofilm communities (Fig. 5 and Table 2). These pathway-involved genes, such as *clpV1* (6.8-fold), *pilA* (17.1-fold) and the *pslBCD* operon, were all significantly upregulated in the microbial biofilm community (*P. aeruginosa* is the dominant species) compared with the planktonic cultures (*P. aeruginosa* is not the dominant species). Our results highlight the biological significance of biofilm formation for *P. aeruginosa* to gain fitness in complex microbial communities,^59^ and we confirmed that H1-T6SS is a powerful weapon for *P. aeruginosa* interspecies competition within the ecological niche in bacterial communities.^39,41^ Confocal imaging analysis for dual-species biofilms also showed that parental strain PAO1 had a higher population than the H1-T6SS-defective mutant Δ*clpV1* when competing against *A. baumannii*, *C. violaceum*, *C. amalonaticus* and *S. maltophilia* while *E. coli* as control (Supplementary Figure 5e–5n). Our findings on *P. aeruginosa* in mixed-species microbial communities are in accordance with previous studies in pure culture or two-species consortia^18,19,26,40^ and suggest that Psl, T4P and H1-T6SS are potential targets to control or eliminate *P. aeruginosa* from complex microbial biofilm communities, such as microbial biofilms on medical devices.

In addition, we demonstrated the sensitivity of the single molecule detection technique, NanoString nCounter® 16 S rRNA array, in monitoring the population dynamics in complex bacterial communities. Our results showed that the accuracy of this technique depends on the specificity and affinity of the probes, which need to be normalised during the calculation of population dynamics.

*P. aeruginosa* can dominate airway infection microbiota^60^ and wound communities after the administration of antibiotics,^61^ suggesting that *P. aeruginosa* has a competitive physiology in mixed-species microbial communities. Our transcriptomic analysis showed that two of the primary mechanisms for *P. aeruginosa* maintaining this competitive advantage are H1-T6SS and biofilm formation. This differs from most previous reports in which many quorum-sensing-regulated virulence factors, such *as* elastase, PQS, pyoverdine and pyocyanin, were used by *P. aeruginosa* to kill other bacteria.^53–57^ The reasons for this difference may be as follows: (1) *P. aeruginosa* *las* and *pqs* systems repress H1-T6SS to compete with other bacteria^58^; (2) quorum-sensing-signalling molecules and virulence factors might function as signals that induce aggressive phenotypes from bacterial competitors as well as hosts via cross-talk, which in turn arrests the growth of *P. aeruginosa*; (3) the high production of quorum-sensing-regulated virulence factors might consume a great deal of energy to reduce the division of *P. aeruginosa* cells or even trigger autolysis phenotypes^62^; and/or (4) the continued fresh medium stream of a flow-tube biofilm cultivation system might wash away the secreted quorum sensing signals and virulence factors and thus reduce the efficacy of quorum sensing. Thus, the cell-contact based T6SS might be the most efficient mechanism for *P. aeruginosa* to outcompete other species. This is supported by our data that loss of ClpV1 in H1-T6SS will decrease the competitive capacity of *P. aeruginosa* in our laboratory model (Fig. 5). Recently, Allsopp et al.^63^ has shown that all three T6SSs in *P. aeruginosa* are more highly expressed at 25°C than at 37°C, however, expressed H2-T6SS and H3-T6SS did not affect *P.* a*eruginosa* competition capacity as much as H1-T6SS at 25°C in our experiment (Fig. 5).

H1-T6SS is well known for its roles in competitiveness and pathogenicity in *P. aeruginosa*.^26,39^ In addition to H1-T6SS, Psl and T4P are critical components of *P. aeruginosa* fitness in the mixed-species biofilm community (Fig. 5). Our result is in accordance with previous research showing that secreted protein A from *S. aureus* can inhibit *P. aeruginosa* biofilm formation by binding to Psl and T4P.^64^ In mixed-species biofilm communities, three *P. aeruginosa*’s most upregulated genes, PA3661, PA2205 and PA1395, are encoding small lipoproteins (Supplementary Table 3). Wood et al.^65^ reported *P. aeruginosa*’s small lipoproteins genes upregulation under cell wall stress, which is regulated by sigma factor AlgU*.* However, expression of *algU* changed less than twofold in our study, suggesting that *P. aeruginosa* may undergoes AlgU-independent stress response in the mixed-species biofilm communities. One limitation of our current study is that we have not elucidated whether *P. aeruginosa* employs T6SS, Psl and T4P sequentially or simultaneously when outcompeting the other species in biofilm communities. In addition, further study is required to validate whether the T6SS, Psl and T4P are required by *P. aeruginosa* to outcompete other species in vivo. Because the amounts of bacteria from in vivo samples are usually low and insufficient for transcriptomic analysis, suitable probes for the NanoString nCounter® array can be explored to examine the population dynamics and gene expression of *P. aeruginosa* from the in vivo communities. Moreover, *K. pneumonia* is the secondary dominant species in our biofilm co-cultures but not in planktonic co-cultures (Fig. 2), and it constantly co-exists with *P. aeruginosa* in vitro and in vivo.^66^ Thus, it would be interesting to ascertain its survival mechanism in future investigations.

## Methods

### Bacterial strains, media and growth conditions

The bacterial species used in this study are listed in Supplementary Table 1. All strains were revived on TSB agar plates and subsequently in TSB liquid medium at 30 °C. Mixed-species communities were cultivated in 10% TSB at 25 °C. To construct *P. aeruginosa* gene knockout mutants and green fluorescent protein (GFP)-tagged strains, AB minimal medium^67^ supplemented with 10 mM citric acid supplemented with appropriate antibiotics was used to select *P. aeruginosa*. *E. coli* was grown in LB medium at 37 °C. In total, 60 µg/ml of gentamicin was used to select *P. aeruginosa*, whereas 15 µg/ml of gentamicin, 10 µg/ml of chloramphenicol and 100 µg/ml of ampicillin were used to select *E. coli*, when appropriate.

### Growth assay and static biofilm cultivation

Overnight cultures of each bacterial species were diluted to OD_600_ of 0.01 in 10% TSB, and 150 µl diluted culture were loaded into 96-well microtitre plates. The plates were incubated in an infinite M200PRO multimode microplate reader (Tecan Schweiz AG, Männedorf, Switzerland) at 25°C, and the OD_600_ of each well was measured and recorded hourly. A 10% TSB blank medium was used as a negative control, and its reading has been subtracted from all the culture readings to plot the growth curves for each species.

To examine the static biofilm-forming capacities of each species, bacterial cultures were prepared using the same procedures as for the growth assay. After overnight cultivation, the planktonic cells were discarded from the microtitre plate, and the wells were washed three times using tap water to remove any residual planktonic cells. A 180 µl volume of 0.1% crystal violet (CV) solution was loaded into each well to stain the biofilm for 15 min. The excess stain in the wells was washed out with tap water three times. Finally, 180 µl of 30% acetate acid was loaded into each well to dissolve CV from the stained biofilm. The optical intensity at OD_550_ was measured by an Infinite M200PRO multimode microplate reader to quantify the biofilms formed by each bacterial species.

### Cultivation of the mixed-species planktonic and biofilm communities

Overnight cultures of the 18 individual bacterial species were diluted in 10% TSB medium and normalised to the same ratio (OD_600_ of each species is ~0.01) to form the initial inoculum for both planktonic cultures in shake flask and biofilm cultures in flow-tube reactors. The planktonic cultures were shaking with 200 rpm at 25°C. The biofilm cultivation was started from initial inoculum attachment to Masterflex^TM^ silicone tube; then fresh 10% TSB medium was continually pumped to flow through these attached cells in silicone tube at 4 ml/h at 25 °C. The 4 ml/h flow will supply enough nutrients for biofilm maturity and take away the planktonic cells in silicone tube. The 24-hour-old planktonic cultures and 5-day-old biofilm cultures were harvested for population dynamic or transcriptomic analysis.

### RNA preparation for sequencing

The bacterial cells were first treated with RNA Protect Reagent (Qiagen®, Germany) to maintain the integrity of RNA. The total RNA was extracted from these bacterial cells using a miRNeasy Mini Kit (Qiagen®, Germany) with modifications. A Turbo DNA-free Kit (Thermo Fisher Scientific®, Lithuania) was used to remove genomic DNA contaminants from total RNA. DNA contamination was assessed with a Qubit® dsDNA High Sensitivity assay (PicoGreen dye) and a Qubit® 2.0 Fluorometer (Invitrogen®, Austria) according to manufacturer’s instructions. Ribosomal RNA was depleted with a Ribo-Zero rRNA removal Kit (Illumina, USA). The integrity of the total RNA was assessed with an Agilent TapeStation System (Agilent Technologies, UK).

The double-stranded coplementary DNAs (cDNAs) were reverse-transcribed using a NEBNext RNA first and second strand synthesis module (NEB®, USA). cDNAs were subjected to Illumina’s TruSeq Stranded mRNA protocol. The quantitated libraries were then pooled at equimolar concentrations and sequenced on an Illumina HiSeq2500 sequencer in rapid mode at a read-length of 100 bp paired-ends.

### NanoString nCounter® population analysis

One nanogram of purified total RNA from the different bacterial cultures was analysed using a NanoString nCounter® Gene Expression CodeSets platform (NanoString Technologies, Inc., United States) with the customised CodeSets for our selected bacterial species (Supplementary Table 1), according to the manufacturer’s protocol, to obtain raw counts. The quality control assessment and raw counts were normalised and analysed using nSolver™ analysis software version 2.6 (NanoString Technologies, Inc., United States). The samples were analysed using the manufacturer-recommended default parameters. The geometric mean was selected for negative control subtraction; if the normalisation factors were outside the range 0.3–3, the normalised factor and the flag lane were computed by the geometric mean. The experiments were performed in triplicate, and the results are presented as the means ± S.D.

### Transcriptomic analysis

The accession number for RNA-seq is SRP128411. The PAO1 genome (NC_002516) was used as the reference for *P. aeruginosa* transcriptomics analysis, with annotation from the Pseudomonas Genome Database (http://www.pseudomonas.com/). The RNA-Seq raw data were analysed using the “RNA-Seq and expression analysis” application in CLC genomics Workbench 10.0 (QIAGEN). The total gene reads from CLC genomics Workbench 10.0 were subjected to the DESeq2 package for statistical analysis^68^ by using R/Bioconductor.^69^ A hierarchical clustering analysis was performed with a negative binomial test using the DESeq2 package. The heatmap.2 package was used to draw a heat map for the differentially expressed genes of *P. aeruginosa* cells with fold change larger than two and an adjusted *p* value smaller than 0.05. The principal component analysis plot was generated in R/Bioconductor.

The adapter-trimmed and assembled RNA sequences of the mixed-species microbial community were used as metatranscriptomics data for analysis. The sequences of the mixed-species communities grown in biofilm and planktonic modes were aligned against the NCBI non-redundant protein database using DIAMOND with default settings. The output aligned gene reads in DAA format were uploaded and ‘meganised’ in MEGAN6.11.1 with a minimum bit-score of 50 and a top percentage of 25.^70^ Functional analyses of these aligned gene reads were performed using SEED classifications. A PCoA was plotted to cluster the samples based on functions. The functional analysis results are illustrated in stacked bar charts.

### Fluorescence-based *P. aeruginosa* population assay

To develop a fast and economical *P. aeruginosa* population dynamics assay in the mixed-species cultures, a single copy of a GFP tag was inserted into the *P. aeruginosa* chromosome by using a mini-Tn7-Gm-gfp transposon.^71^ The other 17 bacterial species without *P. aeruginosa* were collectively used as a background control. The GFP fluorescence readings of both planktonic and biofilm cultures, with gradient proportion of GFP-tagged *P. aeruginosa* derived strains, were recorded using a Tecan infinite M200PRO microplate reader with excitation wavelength 485 nm and emission wavelength 535 nm. The fluorescence-proportion standard curves of each tested strain were generated from these GFP readings (Supplementary Figure 4). The proportions of *P. aeruginosa* derived strains in communities were calculated using these fluorescence-proportion standard curves. All of coefficient of determinations (*R*^2^) of these linear regression lines are larger than 0.97, suggesting that the fluorescence-based population assay is a reliable method to detect *P. aeruginosa* proportions in our mixed-species communities.

### Constructions of *P. aeruginosa* gene deletion and complementation mutants

The upstream fragment of target gene was amplified by primer-1 and primer-2, the downstream fragment of the target gene was amplified by primer-3 and primer-4 (Supplementary Table 4), and then both fragments were fused into a pK18-Gm-*mobsacB* plasmid^72^ and transformed into the *P. aeruginosa* PAO1 wild-type strain to delete the target gene as previously described.^72^ PCR and sequencing were used to confirm the selected mutant strains (Supplementary Table 5).

To complement the *P. aeruginosa clpV1* mutant, a DNA fragment from the *clpV1* upstream 22 bps to the downstream 31 bps was amplified by primers *clpV1*-up and *clpV1*-down (Supplementary Table 4) and double-digested using *Bam*HI and *Hind*III. The purified fragment was ligated with the pUCP22 vector transcribed by a *lacZ* promoter,^73^ which was digested by the same restriction enzymes to construct the pUCP22-*clpV1* complementary plasmid. After sequencing verification, the pUCP22-*clpV1* plasmid was transformed into the *P. aeruginosa* Δ*clpV1* mutant by electroporation to construct a *clpV1* complementation strain.

### Confocal microscopy imaging

*P. aeruginosa* PAO1-GFP-containing planktonic cultures and biofilm cultures (the same samples as those used for the fluorescence-based population assay) were stained with 5 µm SYTO^TM^ 62 Red fluorescent nucleic acid stain (molecular probe) for 15 min to stain all cells before 5 µl of each sample was aliquoted onto a glass slide and covered with a cover slip. For supplementary figure 5, 5 d biofilm cells were scraped from silicone tube into imaging chamber to stain all cells with SYTO^TM^ 62 Red fluorescent nucleic acid stain, because silicone tube is too thick for directly confocal imaging. The samples were visualised and confocal images acquired using confocal laser scanning microscopy (CLSM; Zeiss LSM 780, Carl Zeiss, Germany) with a 63 × 1.40 DICII objective. Green and red fluorescence were observed upon 488 nm and 633 nm excitation wavelengths, respectively.

## Supplementary information

Supplementary figures and tables

## Data Availability

The data that support the findings of this study are available from the corresponding author upon reasonable request. The accession number for RNA-seq is SRP128411.

## Supplementary information

**Supplementary information** accompanies the paper on the *npj Biofilms and Microbiomes* website (10.1038/s41522-018-0076-z).
