# Supplementary material for: Population dynamics and transcriptomic responses of Pseudomonas aeruginosa in a complex laboratory microbial community
Source: NPJ Biofilms Microbiomes. 2019 Jan 14;5:1. doi: 10.1038/s41522-018-0076-z (PMC6334633; doi:10.1038/s41522-018-0076-z)
Supplement: Supplementary file 1 — Supplementary figures and tables [file 41522_2018_76_MOESM1_ESM.pdf]

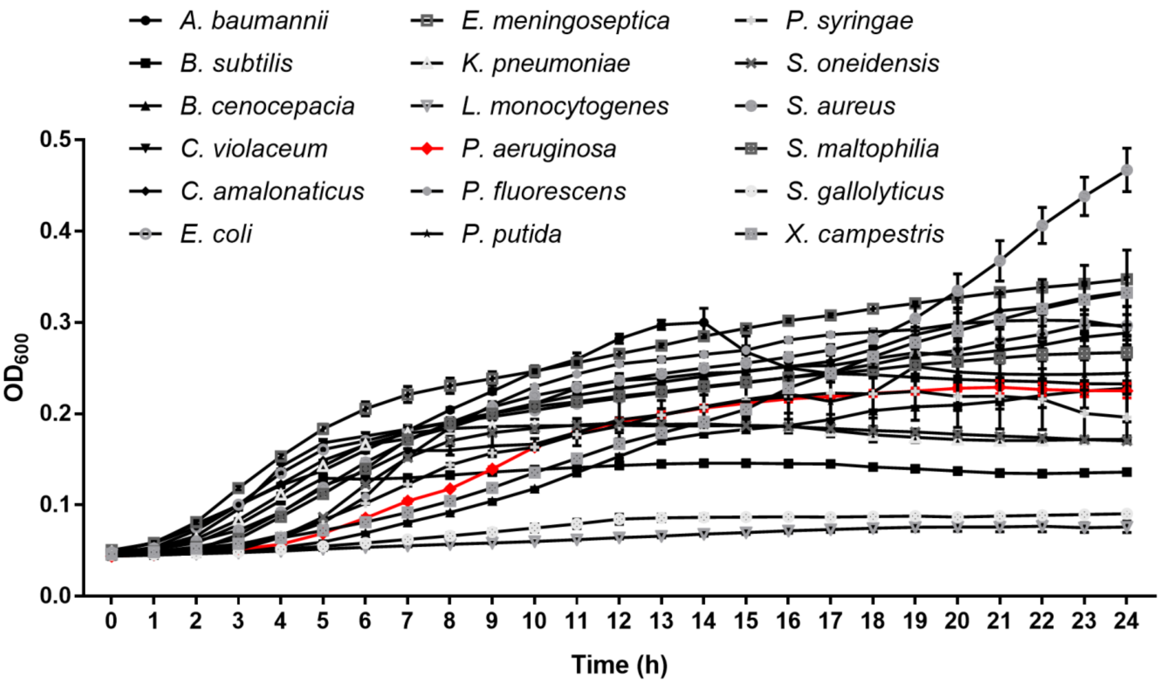

Supplementary Figure 1. Growth curve of the single species contained in the mixed-species microbial community in 10% TSB medium. Overnight culture of each species was diluted to OD_{600} of 0.01 in 10% TSB and loaded into 96-well plates; and the optical density of each species was measured hourly at a wavelength at 600 nm with 5 replicates at 25°C. The background reading of blank medium was deducted from the average value; error bars are showing standard deviations.

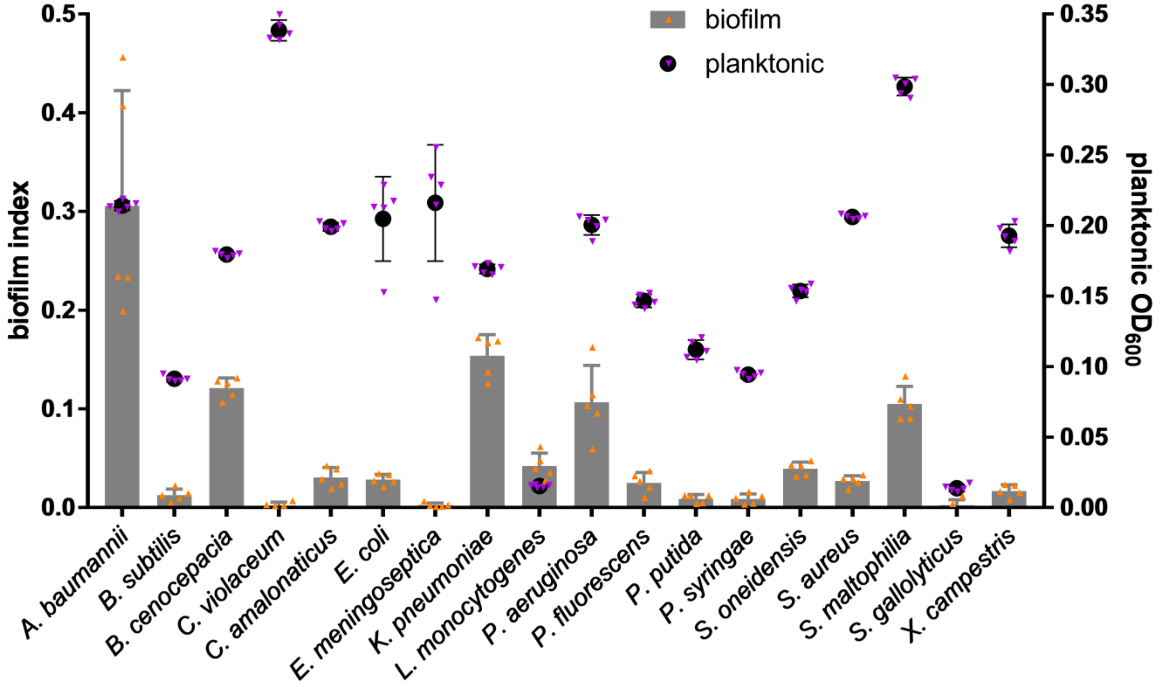

Supplementary Figure 2. Biofilm formation capacity of each species in 10% TSB. The grey columns show each single species biofilm stained by crystal violet after 24 hours of static inoculation at room temperature. The planktonic cells reading is indicated by black circular dot. Each average value was calculated from 5 replicates in dots; error bars are showing standard deviations.

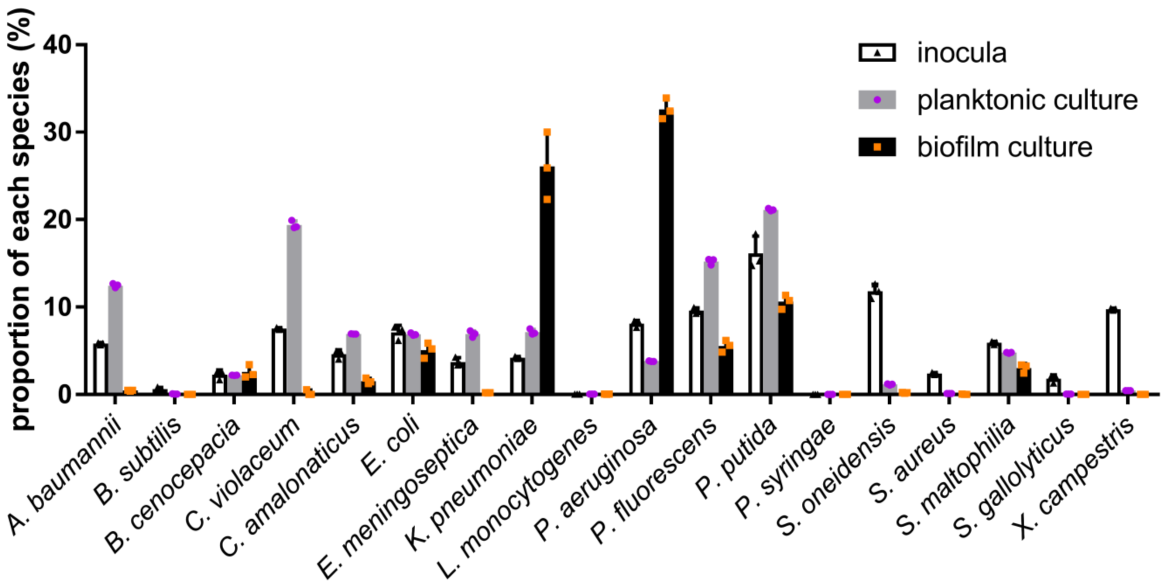

Supplementary Figure 3. Proportion of each species in the mixed-species microbial planktonic (grey) and biofilm (black) communities. The proportion of fourteen species has been normalized using the normalization factor in Table 1 without *L. monocytogenes*, *P. fluorescens*, *P. putida* and *P. syringae*. The inocula is the mixture of all the species mixed at equal OD_{600} amounts at the starting point for both the planktonic and biofilm incubation. Error bars are showing standard deviations of 3 replicates (dots).

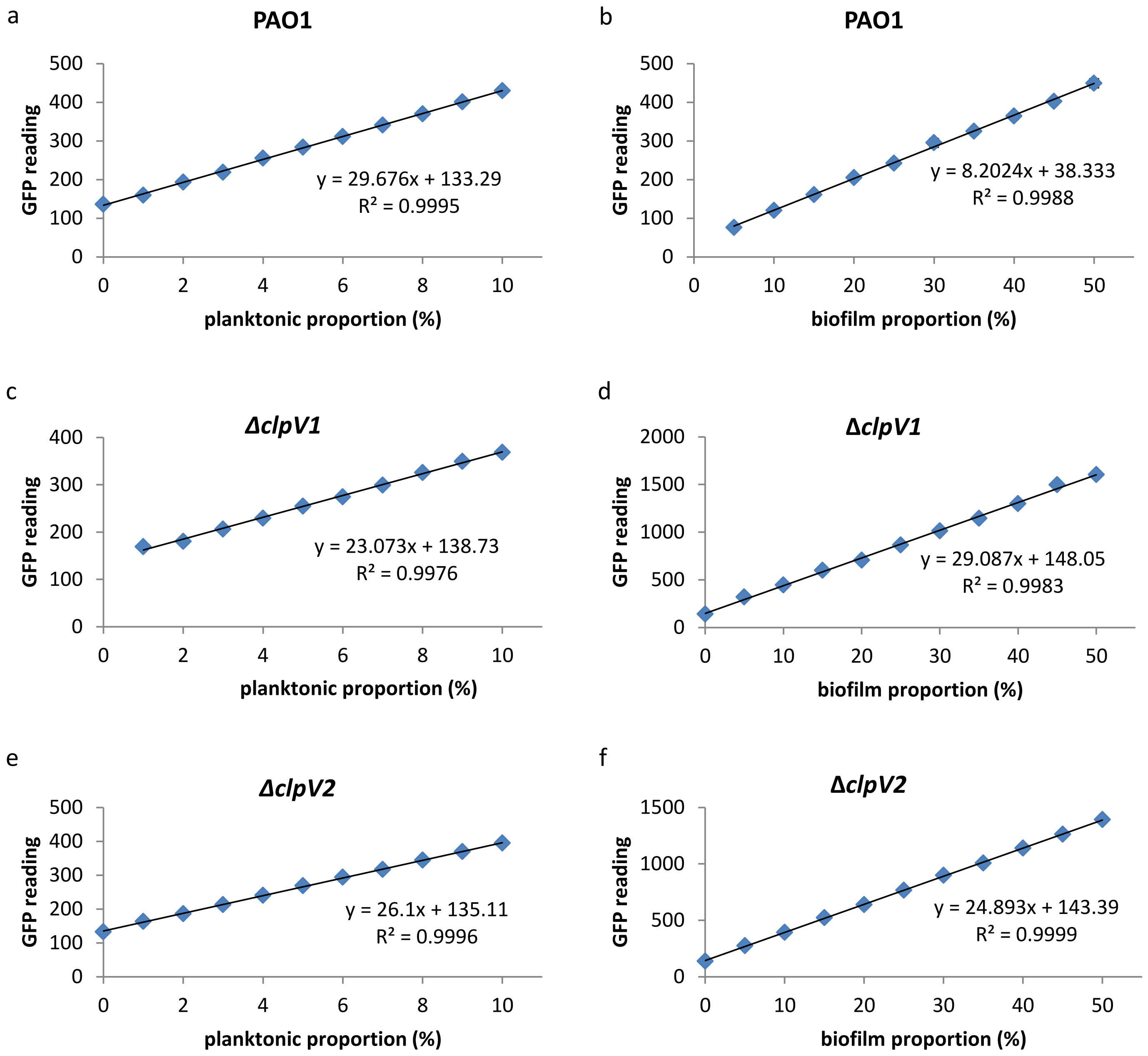
a
PAO1
GFP reading
500
400
300
200
100
0
0
2
4
6
8
10
planktonic proportion (%)
y = 29.676x + 133.29
R² = 0.9995
b
PAO1
GFP reading
500
400
300
200
100
0
0
10
20
30
40
50
biofilm proportion (%)
y = 8.2024x + 38.333
R² = 0.9988
c
ΔclpV1
GFP reading
400
300
200
100
0
0
2
4
6
8
10
planktonic proportion (%)
y = 23.073x + 138.73
R² = 0.9976
d
ΔclpV1
GFP reading
2000
1500
1000
500
0
0
10
20
30
40
50
biofilm proportion (%)
y = 29.087x + 148.05
R² = 0.9983
e
ΔclpV2
GFP reading
500
400
300
200
100
0
0
2
4
6
8
10
planktonic proportion (%)
y = 26.1x + 135.11
R² = 0.9996
f
ΔclpV2
GFP reading
1500
1000
500
0
0
10
20
30
40
50
biofilm proportion (%)
y = 24.893x + 143.39
R² = 0.9999

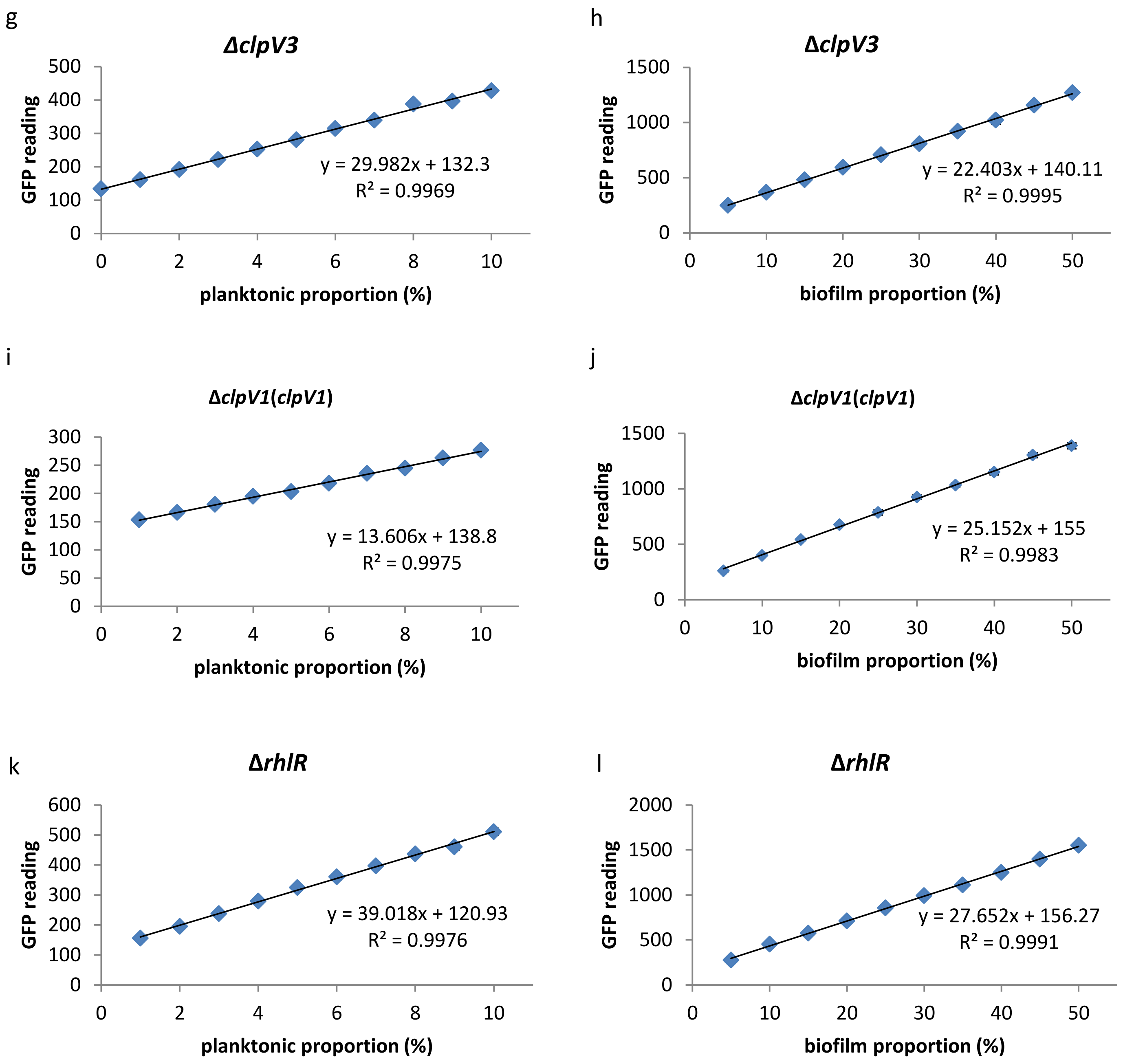

g
ΔclpV3
GFP reading
planktonic proportion (%)
y = 29.982x + 132.3
R² = 0.9969
h
ΔclpV3
GFP reading
biofilm proportion (%)
y = 22.403x + 140.11
R² = 0.9995
i
ΔclpV1(clpV1)
GFP reading
planktonic proportion (%)
y = 13.606x + 138.8
R² = 0.9975
j
ΔclpV1(clpV1)
GFP reading
biofilm proportion (%)
y = 25.152x + 155
R² = 0.9983
k
ΔrhlR
GFP reading
planktonic proportion (%)
y = 39.018x + 120.93
R² = 0.9976
l
ΔrhlR
GFP reading
biofilm proportion (%)
y = 27.652x + 156.27
R² = 0.9991

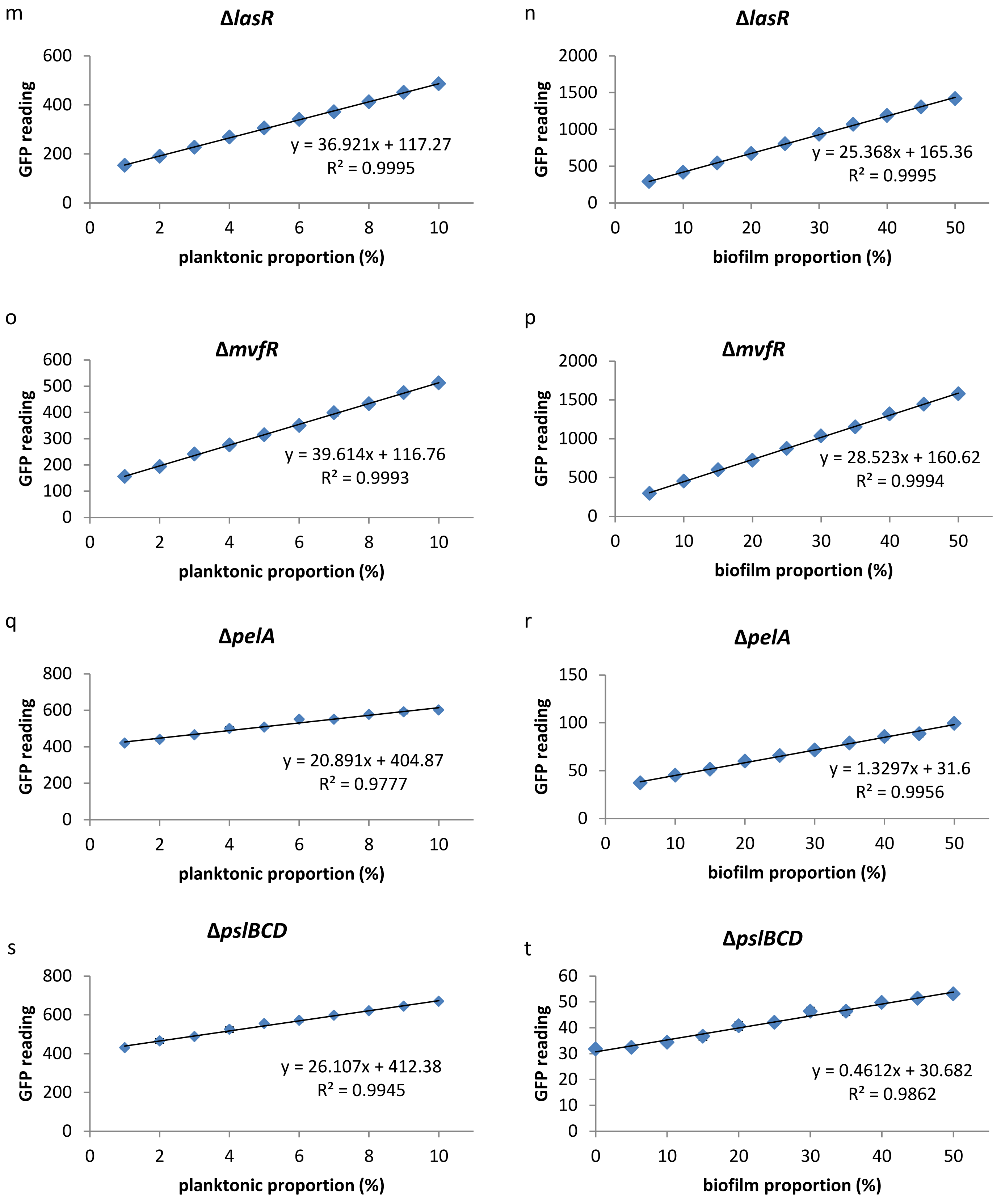

m
ΔlasR
GFP reading
planktonic proportion (%)
y = 36.921x + 117.27
R² = 0.9995
n
ΔlasR
GFP reading
biofilm proportion (%)
y = 25.368x + 165.36
R² = 0.9995
o
ΔmvfR
GFP reading
planktonic proportion (%)
y = 39.614x + 116.76
R² = 0.9993
p
ΔmvfR
GFP reading
biofilm proportion (%)
y = 28.523x + 160.62
R² = 0.9994
q
ΔpelA
GFP reading
planktonic proportion (%)
y = 20.891x + 404.87
R² = 0.9777
r
ΔpelA
GFP reading
biofilm proportion (%)
y = 1.3297x + 31.6
R² = 0.9956
s
ΔpslBCD
GFP reading
planktonic proportion (%)
y = 26.107x + 412.38
R² = 0.9945
t
ΔpslBCD
GFP reading
biofilm proportion (%)
y = 0.4612x + 30.682
R² = 0.9862

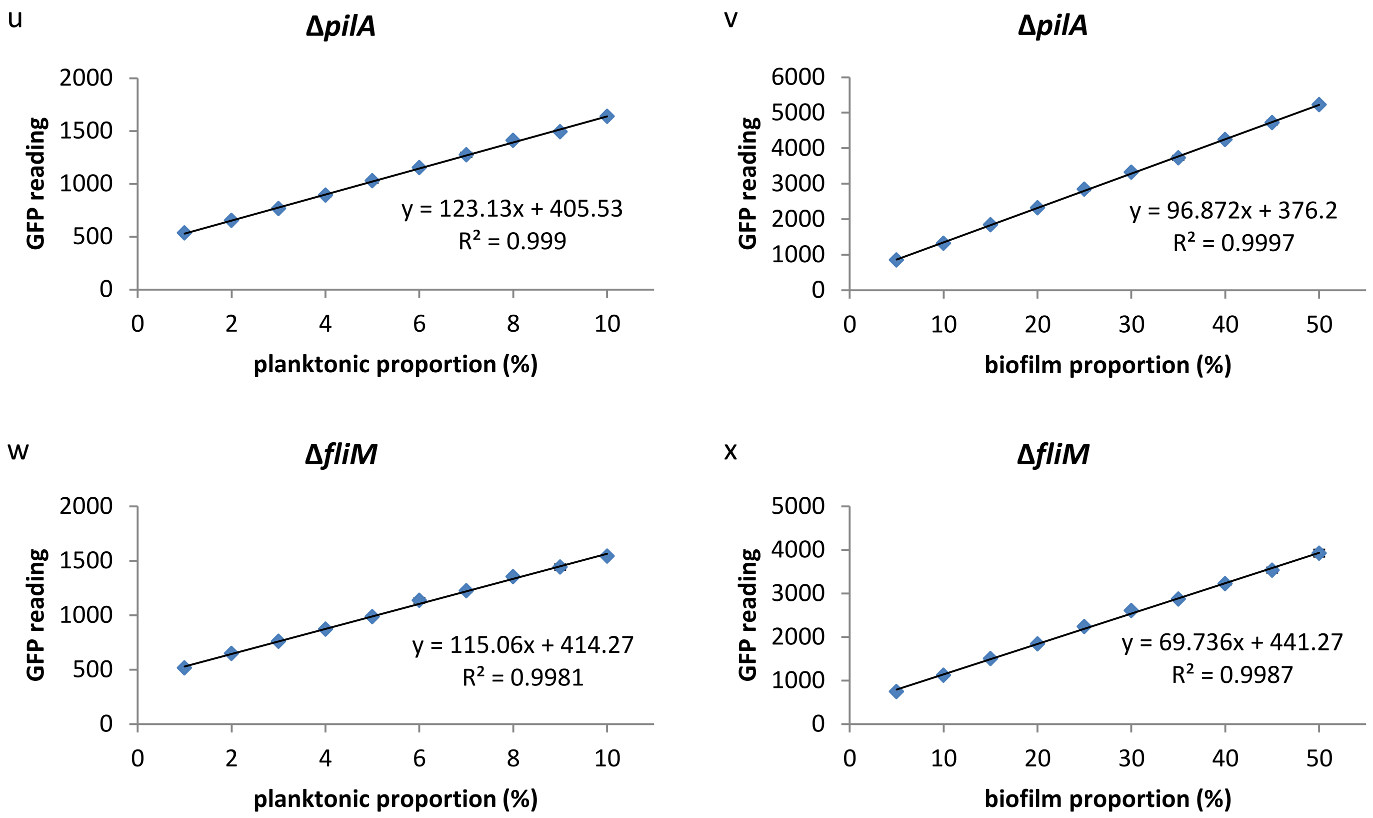

Supplementary Figure 4. Fluorescence-based proportion test standard curves of *P. aeruginosa* parental strain and knockout mutants in mixed-species microbial communities. a, c, e, g, i, k, m, o, q, s, u and w show standard curves for each strain in the planktonic cultures. b, d, f, h, j, l, n, p, r, t, v and x show standard curves of each strain in the biofilm community. The results are presented as the means ± S.D from 3 replicates.

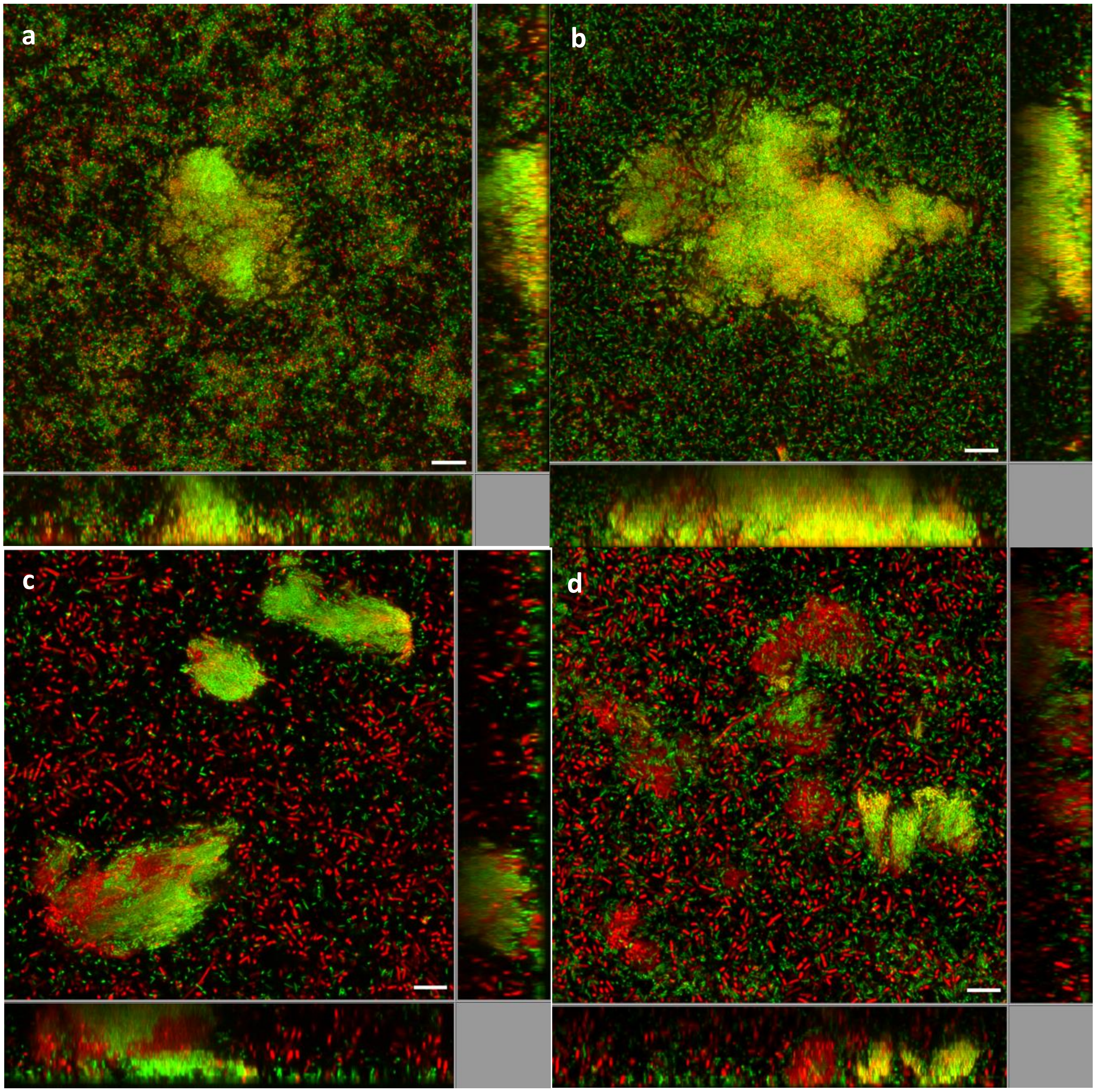
a
b
c
d

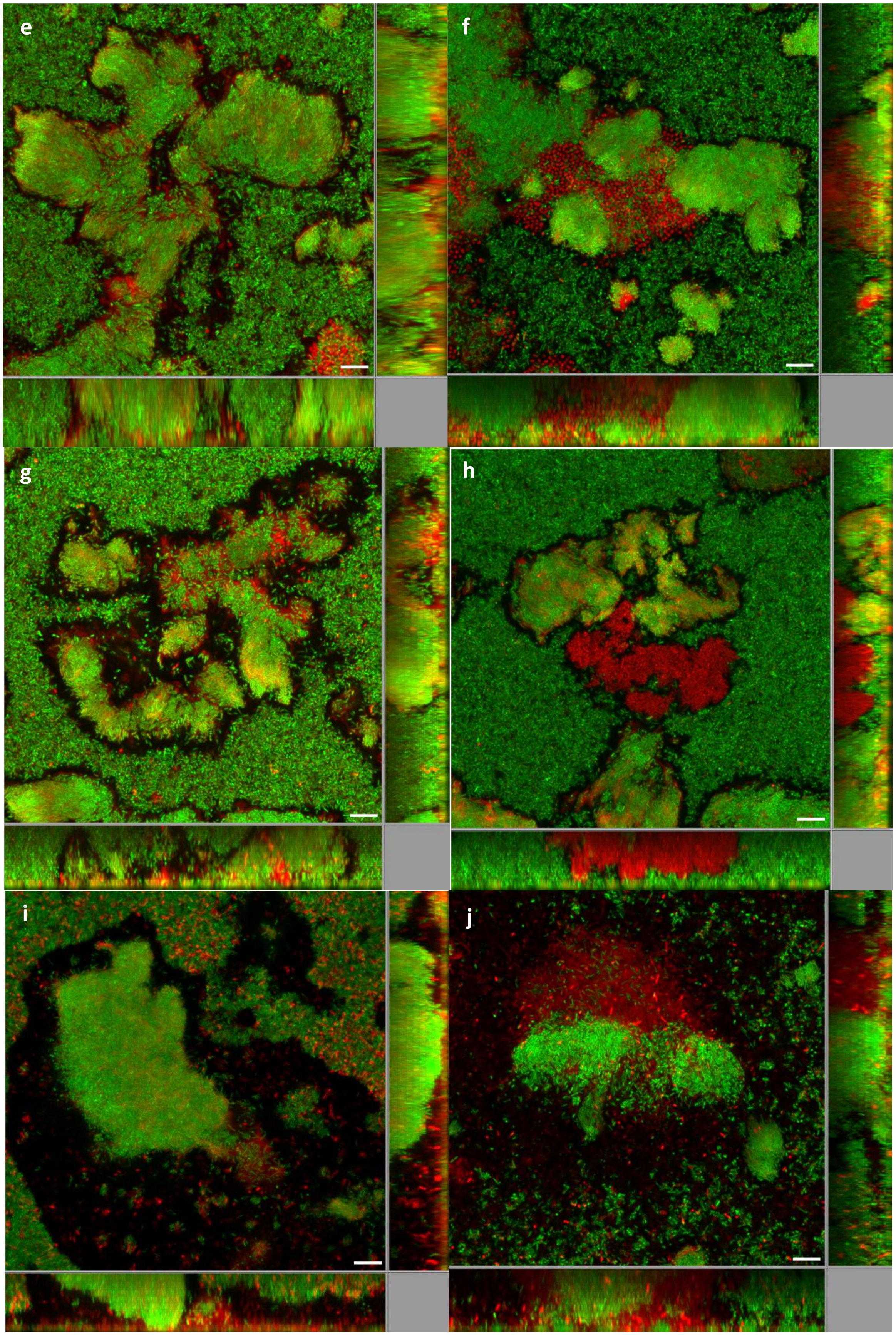
e
f
g
h
i
j

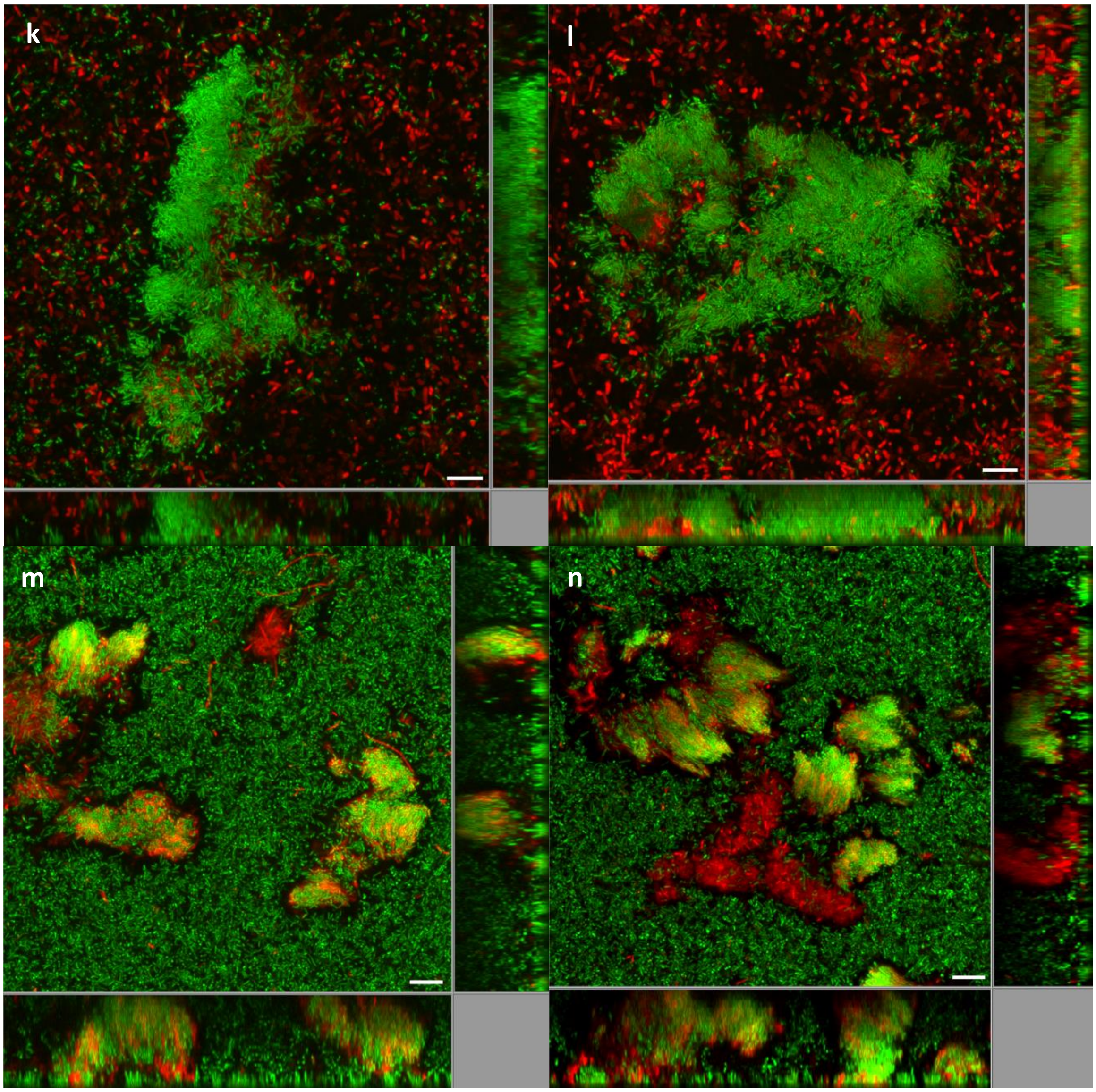

Supplementary Figure 5. Confocal images of biofilm cells. a shows wild type *P. aeruginosa* PAO1 mono-species biofilm; b shows *P. aeruginosa* H1-T6SS mutant *ΔclpV1* mono-species biofilm; c shows PAO1 in 18 species biofilm; d shows *ΔclpV1* in 18 species biofilm; e to n show two species biofilm cells: *P. aeruginosa* PAO1 (e, g, i, k and m) or *ΔclpV1* (f, h, j, l and n) with *A. baumannii* (e and f), *C. violaceum* (g and h), *C. amalonaticus* (i and j), *E. coli* (k and l) and *S. maltophilia* (m and n). GFP-tagged *P. aeruginosa* live cells are in green, all of other live cells and dead cells are in red stained by Syto62. Scale bar represents 10 µm.

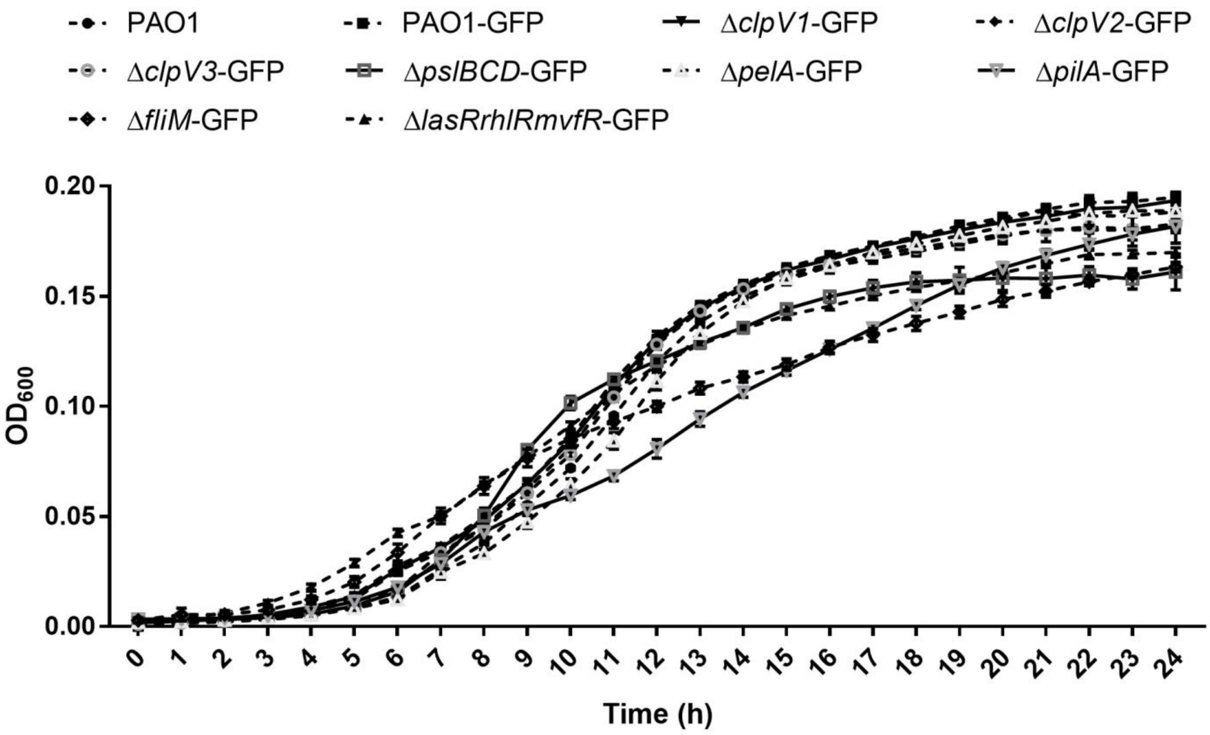

Supplementary Figure 6. Growth curves of the GFP-tagged *P. aeruginosa* parental strain and deletion mutants in 96-well plates under static incubation at room temperature with 6 repetitions.

Supplementary Table 1. Species mixed into microbial community.

Species	16S rRNA array probe sequences	RNA-seq reference
Acinetobacter baumannii ACICU	GCTGTCGTCAGCTCGTGTCGTGAGATGTTGGGTTAAGTCCCGCAACGAGCGCAACCCTT TTCCTTACTTGCCAGCATTTCGGATGGGAACTTTAAGGATA	NC_010611
Bacillus subtilis str. 168	CTGACGGAGCAACGCCGCGTGAGTGATGAAGGTTTTCGGATCGTAAAGCTCTGTTGTTA GGGAAGAACAAGTACCGTTCGAATAGGGCGGTACCTTGACG	NZ_CP010052
Burkholderia cenocepacia J2315	AGTGGGGGATAGCCCGGCGAAAGCCGGATTAATACCGCATACGATCTACGGATGAAAG CGGGGGACCTTCGGGCCTCGCGCTATAGGGTTGGCCGATGGC	NC_011000, NC_011001, NC_011002
Chromobacterium violaceum ATCC12472	GATAGCTCGGCGAAAGCCGGATTAATACCGCATACGCCCTGAGGGGGAAAGCGGGGG ATCGAAAGACCTCGCGTTATACGAGCAGCCGACGTCTGATTAG	NC_005085
Citrobacter amalonaticus Y19	GCAGCCATGCCGCGTGTATGAAGAAGGCCTTCGGGTTGTAAAGTACTTTCAGCGGGGA GGAAGGGATTGTGGTTAATAACCGCAGTCATTGACGTTACCC	NZ_CP011132
Escherichia coli UTI89	GCAGCCATGCCGCGTGTATGAAGAAGGCCTTCGGGTTGTAAAGTACTTTCAGCGGGGA GGAAGGGAGTAAAGTTAATACCTTTGCTCATTGACGTTACCC	NC_007946
Elizabethkingia meningoseptica FMS-007	GGTCCGTAGGCGGACTGATAAGTCAGTGGTGAAATCCGACAGCTTAACTGTCGAACTG CCATTGATACTGTTAGTCTTGAGTAAGGTTGAAGTGGCTGGA	NZ_CP006576
Klebsiella pneumoniae subsp. *pneumoniae* HS11286	GCGTGTGTGAAGAAGGCCTTCGGGTTGTAAAGCACTTTCAGCGGGGAGGAAGGCGTA AGGTTAATAACCTTGTCGATTGACGTTACCCGCAGAAGAAGCA	NC_016845
Listeria monocytogenes EGD-e	AACACGTGGGCAACCTGCCTGTAAGTTGGGGATAACTCCGGGAAACCGGGGCTAATAC CGAATGATAAAGTGTGGCGCATGCCACGCTTTTGAAAGATGG	NC_003210
Pseudomonas aeruginosa PAO1	ATCTGCCTGGTAGTGGGGGATAACGTCCGGAAACGGGCGCTAATACCGCATACGTCCT GAGGGAGAAAGTGGGGGATCTTCGGACCTCACGCTATCAGAT	NC_002516
Pseudomonas fluorescens UK4	CCATGCCGCGTGTGTGAAGAAGGTCTTCGGATTGTAAAGCACTTTAAGTTGGGAGGAA GGGCATTAACCTAATACGTTGGTGTCTTGACGTTACCGACAG	NZ_CP008896
Pseudomonas putida H8234	AGCTTGCTCCTTGATTCAGCGGCGGACGGGTGAGTAATGCCTAGGAATCTGCCTGGTA GTGGGGGACAACGTTTCGAAAGGAACGCTAATACCGCATACG	NC_021491
Pseudomonas syringae pv. *tomato* str. DC3000	GGAGCATGTGGTTTAATTCGAAGCAACGCGAAGAACCTTACCAGGCCTTGACATCCAAT GAATCCTTTAGAGATAGAGGAGTGCCTTCGGGAGCATTGAG	NC_004578

Shewanella oneidensis MR-1	AAGCGTGGGGAGCAAACAGGATTAGATACCCTGGTAGTCCACGCCGTAAACGATGTCT ACTCGGAGTTTGGTGTCTTGAACACTGGGCTCTCAAGCTAAC	NC_004347
Staphylococcus aureus subsp. *aureus* NCTC8325	AAATCATCATGCCCCTTATGATTTGGGCTACACACGTGCTACAATGGACAATACAAAGG GCAGCGAAACCGCGAGGTCAAGCAAATCCCATAAAGTTGTT	NC_007795
Stenotrophomonas maltophilia K279a	CAGCCATACCGCGTGGGTGAAGAAGGCCTTCGGGTTGTAAAGCCCTTTTGTTGGGAAA GAAATCCAGCTGGCTAATACCCGGTTGGGATGACGGTACCCA	NC_010943
Streptococcus gallolyticus subsp. *Gallolyticus* ATCC43143	GAGCGTTGTCCGGATTTATTGGGCGTAAAGCGAGCGCAGGCGGTTTAATAAGTCTGAA GTTAAAGGCAGTGGCTTAACCATTGTTCGCTTTGGAAACTGT	NC_017576
Xanthomonas campestris pv. *campestris* str. 8004	ATCCAGCCATGCCGCGTGGGTGAAGAAGGCCTTCGGGTTGTAAAGCCCTTTTGTTGGG AAAGAAAAGCAGTCGGTTAATACCCGATTGTTCTGACGGTAC	NC_007086

Supplementary Table 2. Normalized 16S rRNAs percentage in each group (unit: %).

	Input	planktonic	biofilm
A. baumannii	5.81±0.12	12.47±0.22	0.44±0.07
B. subtilis	0.61±0.22	0.06±0.00	0.01±0.00
B. cenocepacia	2.29±0.54	2.20±0.03	2.58±0.74
C. violaceum	7.54±0.11	19.40±0.45	0.19±0.32
C. amalonaticus	4.61±0.50	6.93±0.03	1.52±0.31
E. coli	7.10±0.80	6.90±0.14	5.09±0.88
E. meningoseptica	3.71±0.51	6.93±0.37	0.20±0.01
K. pneumoniae	4.22±0.13	7.14±0.31	26.08±3.85
P. aeruginosa	8.11±0.37	3.81±0.04	32.62±1.19
S. oneidensis	11.81±0.82	1.16±0.06	0.21±0.04
S. aureus	2.38±0.13	0.13±0.00	0.03±0.01
S. maltophilia	5.91±0.18	4.79±0.05	3.03±0.50
S. gallolyticus	1.82±0.45	0.05±0.00	0.00±0.00
X. campestris	9.76±0.11	0.45±0.01	0.00±0.00

Supplementary Table 3. Up and down regulated genes in mixed-species biofilm community compared with mono species biofilm of *P. aeruginosa*.

Locus tag	Gene name	Fold change	Adjust p value	Product	Pathway
PA3661	PA3661	46.8	6.39E-35		
PA0989	PA0989	31.3	3.51E-44		
PA2205	PA2205	17.79	2.35E-38		
PA1395	PA1395	15.11	7.03E-17		
PA0941	PA0941	13.69	7.34E-32		
PA0940	PA0940	13.41	5.71E-40		
PA2285	PA2285	13.01	1.81E-12		
PA4156	PA4156	10.87	6.08E-24	FvbA	
PA0939	PA0939	10.3	7.38E-14		
PA1254	PA1254	10.14	3.21E-28	delta1-pyrroline-4-hydroxy-2-carboxylate deaminase, LphC	Arginine and proline metabolism
PA1256	PA1256	8.22	5.06E-14	ABC transporter ATP-binding protein, LhpO	
PA0441	*dht*	7.43	2.49E-11	dihydropyrimidinase	beta-Alanine metabolism
PA0042	PA0042	7.28	5.25E-23		
PA2550	PA2550	7.16	1.51E-18	probable acyl-CoA dehydrogenase	beta-Alanine metabolism
PA1394	PA1394	6.93	2.90E-07		
PA4623	PA4623	6.04	3.51E-10		
PA1253	PA1253	5.41	1.49E-06	alpha-ketoglutaric semialdehyde dehydrogenase, LhpG	Ascorbate and aldarate metabolism
PA0083	*tssB1*	5.34	3.43E-21	TssB1	type VI secretion system
PA0084	*tssC1*	5.24	5.74E-29	TssC1	type VI secretion system
PA0090	*clpV1*	4.96	2.43E-19	ClpV1	type VI secretion system
PA5181	PA5181	4.84	1.77E-24		
PA0085	*hcp1*	4.82	2.96E-49	Hcp1	type VI secretion system
PA1195	PA1195	4.6	1.66E-46	dimethylarginine dimethylaminohydrolase DdaH	Arginine and proline metabolism
PA1194	PA1194	4.57	6.59E-38		
PA1216	PA1216	4.54	1.43E-05		

PA1178	*oprH*	4.52	6.48E-20	PhoP/Q and low Mg2+ inducible outer membrane protein H1 precursor	
PA4893	*ureG*	4.51	1.47E-16	urease accessory protein UreG	urea degradation II
PA2792	PA2792	4.31	4.58E-06		
PA4155	PA4155	4.27	1.67E-04		
PA5020	PA5020	4.23	2.71E-10	probable acyl-CoA dehydrogenase	Biosynthesis of secondary metabolites
PA0263	*hcpC*	4.23	1.51E-16	secreted protein Hcp	type VI secretion system
PA5180	PA5180	4.22	4.93E-11		
PA4891	*ureE*	4.19	2.85E-08	urease accessory protein UreE	urea degradation II
PA2918	PA2918	4.14	1.36E-06		
PA1255	PA1255	4.08	1.38E-05	D-hydroxyproline epimerase, LhpK	
PA1218	PA1218	3.96	1.42E-04		
PA1217	PA1217	3.95	2.12E-05	probable 2-isopropylmalate synthase	2-Oxocarboxylic acid metabolism
PA2312a	PA2312a	3.94	5.52E-10		
PA0444	PA0444	3.88	1.30E-04	N-carbamoyl-beta-alanine amidohydrolase	Metabolic pathways
PA1970	PA1970	3.86	7.48E-04		
PA2365	*tssB3*	3.78	1.15E-19	TssB3	type VI secretion system
PA2366	*tssC3*	3.75	8.11E-35	TssC3	type VI secretion system
PA2793	PA2793	3.72	4.48E-10		
PA0099	PA0099	3.61	2.10E-13		
PA4726.11	*crcZ*	3.58	4.94E-09		
PA1410	PA1410	3.55	2.35E-03	probable periplasmic spermidine/putrescine-binding protein	ABC transporters
PA0091	*vgrG1*	3.53	8.63E-11	VgrG1	type VI secretion system
PA1512	*hcpA*	3.52	1.34E-12	secreted protein Hcp	type VI secretion system
PA1942	PA1942	3.47	6.44E-06		
PA4531	PA4531	3.43	1.26E-08		
PA1343	PA1343	3.4	1.38E-05		
PA5098	*hutH*	3.4	2.63E-06	histidine ammonia-lyase	Histidine metabolism
PA0439	PA0439	3.38	1.30E-02	probable oxidoreductase	beta-Alanine metabolism
PA1634	*kdpB*	3.37	1.26E-07	potassium-transporting ATPase, B chain	Two-component system

PA1416	PA1416	3.31	4.63E-03	conserved hypothetical protein	Amino sugar and nucleotide sugar metabolism
PA1507	PA1507	3.3	2.87E-03		
PA1666	PA1666	3.3	1.22E-09	Lip2	type VI secretion system
PA1214	PA1214	3.29	1.02E-02		
PA4211	*phzB1*	3.29	6.56E-05	probable phenazine biosynthesis protein	Phenazine biosynthesis
PA2274	PA2274	3.28	3.73E-03		
PA4221	*fptA*	3.23	9.44E-10	Fe(III)-pyochelin outer membrane receptor precursor	
PA1260	PA1260	3.23	2.53E-04	ABC transporter periplasmic-binding protein, LhpP	
PA1669	*icmF2*	3.21	1.21E-12	IcmF2	type VI secretion system
PA1602	PA1602	3.19	4.21E-09	probable oxidoreductase	Microbial metabolism in diverse environments
PA0840	PA0840	3.18	2.97E-08		
PA0160	PA0160	3.16	4.68E-03		
PA5097	PA5097	3.14	6.91E-04		
PA2759	PA2759	3.13	7.54E-08		
PA3291	PA3291	3.12	6.80E-07		
PA5383	PA5383	3.08	5.82E-03		
PA2367	PA2367	3.08	1.51E-16	Hcp3	type VI secretion system
PA1657	*tssB2*	3.06	7.09E-16	TssB2	type VI secretion system
PA2282	PA2282	3.04	1.26E-03		
PA5159	PA5159	3.03	4.92E-05		
PA1864	PA1864	3.02	1.98E-04		
PA0132	PA0132	3.01	4.35E-04	Beta-alanine:pyruvate transaminase	Beta-alanine catabolism
PA2298	PA2298	2.99	2.95E-08		
PA4022	PA4022	2.97	7.14E-09	hydrazone dehydrogenase, HdhA	Biosynthesis of secondary metabolites
PA0050	PA0050	2.95	1.96E-16		
PA3334	PA3334	2.95	1.65E-18		
PA0508	PA0508	2.95	4.88E-11		
PA5167	PA5167	2.95	4.96E-11	DctP	C(4)-dicarboxylate transport
PA0563	PA0563	2.95	3.35E-09		

PA1967	PA1967	2.93	1.85E-09		
PA2041	PA2041	2.92	6.15E-03	Amino acid permease	Polyamine catabolism
PA1187	PA1187	2.9	1.28E-07	probable acyl-CoA dehydrogenase	beta-Alanine metabolism
PA5172	*arcB*	2.9	7.47E-25	ornithine carbamoyltransferase, catabolic	Arginine and proline metabolism
PA2917	PA2917	2.9	6.17E-06		
PA2204	PA2204	2.89	1.77E-02		
PA1737	PA1737	2.87	9.00E-25	probable 3-hydroxyacyl-CoA dehydrogenase	alpha-Linolenic acid metabolism
PA1844	PA1844	2.86	1.41E-02	Tse1	type VI secretion system
PA0100	PA0100	2.86	6.25E-13		
PA0088	*tssF1*	2.85	1.47E-05	TssF1	type VI secretion system
PA1183	*dctA*	2.83	4.45E-07	C4-dicarboxylate transport protein	C(4)-dicarboxylate transport
PA1179	*phoP*	2.83	6.53E-10	two-component response regulator PhoP	Cationic antimicrobial peptide (CAMP) resistance
PA3058	*pelG*	2.82	1.56E-02	PelG	Biofilm formation
PA1845	PA1845	2.81	5.10E-03		
PA1667	*tssJ2*	2.8	1.41E-06	TssJ2	type VI secretion system
PA1601	PA1601	2.8	5.46E-08		
PA1267	PA1267	2.8	1.04E-09	D-hydroxyproline dehydrogenase beta-subunit, LphB	Arginine and proline metabolism
PA1510	PA1510	2.8	8.53E-11	type 6 PGAP1-like effector, TplE	
PA3972	PA3972	2.79	3.01E-10		
PA4630	PA4630	2.78	1.68E-04		
PA1665	PA1665	2.74	1.01E-07		
PA1791	PA1791	2.74	1.32E-04		
PA0529	PA0529	2.74	3.54E-05		
PA1419	PA1419	2.73	4.26E-03		
PA4153	PA4153	2.73	2.81E-02	2,3-butanediol dehydrogenase	Butanoate metabolism
PA3229	PA3229	2.71	2.02E-04		
PA4881	PA4881	2.7	1.90E-06		
PA0323	PA0323	2.68	2.89E-02	probable binding protein component of ABC transporter	ABC transporters
PA4205	*mexG*	2.67	7.40E-03	hypothetical protein	Quorum sensing
PA4892	*ureF*	2.66	1.46E-02	urease accessory protein UreF	urea degradation II
PA0070	PA0070	2.66	4.16E-08	TagQ1	type VI secretion system

PA2536	PA2536	2.66	8.85E-06	probable phosphatidate cytidylyltransferase	Biosynthesis of secondary metabolites
PA1658	*tssC2*	2.65	9.47E-15	TssC2	type VI secretion system
PA1557	*ccoN2*	2.65	3.67E-14	Cytochrome c oxidase, cbb3-type, CcoN subunit	Metabolic pathways
PA5297	*poxB*	2.65	7.28E-07	pyruvate dehydrogenase (cytochrome)	Pyruvate metabolism
PA5173	*arcC*	2.64	3.81E-25	carbamate kinase	allantoin degradation IV (anaerobic)
PA5267	*hcpB*	2.64	2.90E-07	secreted protein Hcp	type VI secretion system
PA3841	*exoS*	2.62	4.21E-04	exoenzyme S	Type III Secretion
PA0260	PA0260	2.62	2.48E-15	Tle3	
PA0089	PA0089	2.62	8.15E-03	TssG1	type VI secretion system
PA1663	PA1663	2.61	1.48E-06		
PA0507	PA0507	2.61	4.31E-03		
PA2634	*aceA*	2.59	1.53E-08	isocitrate lyase AceA	Biosynthesis of secondary metabolites
PA5171	*arcA*	2.59	5.26E-21	arginine deiminase	arginine/ornithine antiporter
PA2574	*alkB1*	2.55	5.82E-03	alkane-1-monooxygenase	aliphatic compound catabolism
PA1633	*kdpA*	2.54	2.56E-05	potassium-transporting ATPase, A chain	Two-component system
PA3484	PA3484	2.54	6.03E-04	Tse3	type VI secretion system
PA3729	PA3729	2.54	9.60E-13		
PA2664	*fhp*	2.54	4.16E-03	flavohemoprotein	
PA0898	*aruD*	2.53	3.54E-05	N-Succinylglutamate 5-semialdehyde dehydrogenase	Arginine and proline metabolism
PA5542	PA5542	2.53	4.53E-04		
PA0459	PA0459	2.51	2.78E-17		
PA2101	PA2101	2.5	3.81E-02		
PA0130	PA0130	2.5	2.77E-03	3-Oxopropanoate dehydrogenase	Beta-alanine catabolism
PA1649	PA1649	2.48	1.21E-02		
PA0097	PA0097	2.48	9.68E-04		
PA3292	PA3292	2.47	2.84E-03		
PA4139	PA4139	2.46	8.44E-06		
PA2540	PA2540	2.46	2.60E-06		
PA4582	PA4582	2.45	1.49E-02		
PA1600	PA1600	2.45	2.06E-04		

PA3059	*pelF*	2.44	1.96E-02	PelF	Biofilm formation
PA4218	PA4218	2.44	1.15E-05		
PA1180	*phoQ*	2.43	3.35E-06	two-component sensor PhoQ	Cationic antimicrobial peptide (CAMP) resistance
PA1432	*lasI*	2.43	8.33E-13	autoinducer synthesis protein LasI	Biofilm formation
PA0086	PA0086	2.43	5.03E-03	TagJ1	type VI secretion system
PA0077	*icmF1*	2.43	6.35E-06	IcmF1	type VI secretion system
PA1673	PA1673	2.42	1.22E-09		
PA0291	*oprE*	2.42	7.82E-14		
PA3250	PA3250	2.41	9.68E-10	hypothetical protein	Quorum sensing
PA2040	PA2040	2.41	7.93E-04	Glutamylpolyamine synthetase	Alanine, aspartate and glutamate metabolism
PA3441	PA3441	2.41	1.05E-02		
PA3363	*amiR*	2.4	2.75E-06	aliphatic amidase regulator	
PA1856	PA1856	2.4	2.16E-02	probable cytochrome oxidase subunit	Metabolic pathways
PA0092	PA0092	2.4	7.91E-03		
PA3728	PA3728	2.39	9.41E-08		
PA2490	PA2490	2.38	7.27E-03		
PA1662	PA1662	2.36	7.24E-09	clpV2	type VI secretion system
PA1661	tssH2	2.35	3.12E-05	TssH2	type VI secretion system
PA0277	PA0277	2.34	2.36E-02		
PA0261	PA0261	2.34	3.37E-04		
PA1556	ccoO2	2.34	7.04E-13	Cytochrome c oxidase, cbb3-type, CcoO subunit	Metabolic pathways
PA2889	*atuD*	2.34	8.77E-03	citronellyl-CoA dehydrogenase, AtuD	Acylic monoterpene utilization
PA3427	PA3427	2.34	1.56E-04		
PA3277	PA3277	2.33	1.62E-04		
PA1941	PA1941	2.32	1.36E-03		
PA1648	PA1648	2.32	7.80E-04		
PA0041	PA0041	2.31	4.81E-10		
PA4395	PA4395	2.31	1.37E-07		
PA5410	*gbcA*	2.3	2.17E-02	GbcA	Aromatic compound catabolism
PA2826	PA2826	2.29	3.69E-05	probable glutathione peroxidase	Arachidonic acid metabolism

PA4489	PA4489	2.28	6.05E-10		
PA0896	*aruF*	2.28	3.22E-05	subunit I of arginine N2-succinyltransferase = ornithine N2-succinyltransferase	Arginine and proline metabolism
PA4435	PA4435	2.28	1.15E-05	probable acyl-CoA dehydrogenase	beta-Alanine metabolism
PA1668	PA1668	2.27	1.92E-04	DotU2	type VI secretion system
PA2537	PA2537	2.27	1.60E-03		
PA0082	*tssA1*	2.27	5.37E-03	TssA1	type VI secretion system
PA1555	*ccoP2*	2.27	1.62E-13	Cytochrome c oxidase, cbb3-type, CcoP subunit	Metabolic pathways
PA1708	*popB*	2.27	4.07E-03	translocator protein PopB	Type III Secretion
PA1079	*flgD*	2.26	1.03E-08	flagellar basal-body rod modification protein FlgD	Flagella assembly
PA3327	PA3327	2.26	1.21E-05	probable non-ribosomal peptide synthetase	fatty acid biosynthesis
PA5169	PA5169	2.26	1.83E-03	DctM	C(4)-dicarboxylate transport
PA1418	PA1418	2.26	6.06E-03		
PA1828	PA1828	2.25	1.42E-04		
PA1555	*ccoQ2*	2.25	2.01E-10	Cytochrome c oxidase, cbb3-type, CcoP subunit	Metabolic pathways
PA4228	*pchD*	2.24	1.92E-04	pyochelin biosynthesis protein PchD	Biosynthesis of siderophore group nonribosomal peptides
PA5421	*fdhA*	2.24	2.91E-02	glutathione-independent formaldehyde dehydrogenase	Carbon metabolism
PA4328	PA4328	2.24	1.37E-08		
PA1797a	PA1797a	2.22	2.99E-02		
PA1842	PA1842	2.22	2.57E-03		
PA0897	*aruG*	2.22	1.50E-04	subunit II of arginine N2-succinyltransferase = ornithine N2-succinyltransferase	Arginine and proline metabolism
PA3332	PA3332	2.21	2.53E-08		
PA3179	PA3179	2.21	6.91E-05		
PA3232	PA3232	2.2	2.15E-02	probable nuclease	DNA replication
PA5096	PA5096	2.19	4.63E-03	probable binding protein component of ABC transporter	ABC transporters
PA1290	PA1290	2.19	1.11E-03		
PA5227	*ssrS*	2.18	1.32E-04		
PA1659	PA1659	2.18	1.09E-03		type VI secretion system
PA3294	PA3294	2.17	9.33E-03	VgrG4a	type VI secretion system
PA4236	*katA*	2.15	8.31E-15	catalase	Biosynthesis of antibiotics

PA5208	PA5208	2.15	1.40E-08		
PA5026	PA5026	2.15	8.55E-05		
PA2575	PA2575	2.15	1.01E-05		
PA4506	PA4506	2.14	3.80E-05	dipeptide ABC transporter ATP-binding protein DppF	ABC transporters
PA5093	PA5093	2.14	5.29E-03	probable histidine/phenylalanine ammonia-lyase	Histidine metabolism
PA0495	PA0495	2.14	4.20E-02		
PA2009	*hmgA*	2.14	4.97E-03	homogentisate 1,2-dioxygenase	Aromatic compound catabolism
PA3727	PA3727	2.13	6.33E-03		
PA1168	PA1168	2.13	2.20E-03		
PA4542	*clpB*	2.13	7.35E-11	ClpB	
PA2539	PA2539	2.13	2.25E-02		
PA1951	PA1951	2.13	1.93E-04		
PA4587	*ccpR*	2.13	5.59E-08		
PA1509	PA1509	2.12	4.47E-06		
PA4490	PA4490	2.12	1.56E-03		
PA0779	PA0779	2.12	6.47E-09		
PA2371	PA2371	2.11	3.23E-07	ClpV3	type VI secretion system
PA4140	PA4140	2.11	2.26E-02	hypothetical protein	Amino sugar and nucleotide sugar metabolism
PA0280	*cysA*	2.11	4.84E-02	sulfate transport protein CysA	ABC transporters
PA4147	*acoR*	2.1	2.83E-04	transcriptional regulator AcoR	
PA4230	*pchB*	2.09	2.49E-03	salicylate biosynthesis protein PchB	Biosynthesis of antibiotics
PA5094	PA5094	2.08	1.54E-03	probable ATP-binding component of ABC transporter	ABC transporters
PA1078	*flgC*	2.08	1.88E-04	flagellar basal-body rod protein FlgC	Flagella assembly
PA3329	PA3329	2.08	1.67E-04		
PA0079	*tssK1*	2.06	4.38E-03	TssK1	type VI secretion system
PA0074	*ppkA*	2.06	2.15E-03	serine/threonine protein kinase PpkA	type VI secretion system
PA4463	PA4463	2.06	7.73E-08		
PA5095	PA5095	2.05	4.42E-03	probable permease of ABC transporter	ABC transporters
PA4443	*cysD*	2.05	3.52E-02	ATP sulfurylase small subunit	Biosynthesis of antibiotics
PA0776	PA0776	2.04	8.40E-06		
PA1837	PA1837	2.04	4.77E-02		

PA1546	*hemN*	2.04	2.00E-08	oxygen-independent coproporphyrinogen III oxidase	heme biosynthesis II (anaerobic)
PA1736	PA1736	2.04	3.58E-07	probable acyl-CoA thiolase	Benzoate degradation
PA5170	*arcD*	2.03	2.33E-08	arginine/ornithine antiporter	
PA3021	PA3021	2.02	3.03E-04		
PA2369	PA2369	2	1.33E-03		
PA5012	*waaF*	-2	2.40E-02	heptosyltransferase II	Lipopolysaccharide biosynthesis
PA3347	PA3347	-2.01	3.68E-05	HptB-dependent secretion and biofilm anti anti-sigma factor HsbA	Biofilm formation
PA0572	PA0572	-2.03	1.05E-12		
PA4869	PA4869	-2.04	2.34E-02		
PA2782	PA2782	-2.05	4.35E-03		
PA4131	PA4131	-2.05	4.75E-05		
PA1784	PA1784	-2.06	2.37E-05		
PA3563	*fruR*	-2.07	4.42E-02		
PA3875	*narG*	-2.07	2.00E-03	respiratory nitrate reductase alpha chain	Microbial metabolism in diverse environments
PA3231	PA3231	-2.07	2.84E-02		
PA3460	PA3460	-2.08	4.26E-03		
PA0571	PA0571	-2.08	4.07E-03		
PA3877	*narK1*	-2.08	2.46E-03	nitrite extrusion protein 1	Nitrogen metabolism
PA2587	*pqsH*	-2.09	4.85E-04	probable FAD-dependent monooxygenase	2-heptyl-3-hydroxy-4(1H)-quinolone biosynthesis
PA4621	PA4621	-2.09	7.20E-04		
PA0524	*norB*	-2.09	1.27E-03	nitric-oxide reductase subunit B	Denitrification
PA1689	PA1689	-2.1	2.84E-03	conserved hypothetical protein	Glycerophospholipid metabolism
PA3102	*xcpS*	-2.1	5.91E-03	general secretion pathway protein F	Bacterial secretion system
PA1686	*alkA*	-2.11	3.38E-02	DNA-3-methyladenine glycosidase II	Base excision repair
PA1137	PA1137	-2.11	1.04E-02	probable oxidoreductase	alpha-Linolenic acid metabolism
PA2391	*opmQ*	-2.13	2.38E-02		
PA4027	PA4027	-2.14	4.16E-05		
PA4847	*accB*	-2.14	1.53E-03	biotin carboxyl carrier protein (BCCP)	Biosynthesis of antibiotics
PA3880	PA3880	-2.14	7.28E-05		

PA2527	PA2527	-2.15	1.09E-03	MuxB	Two-component system
PA3283	PA3283	-2.17	6.29E-03		
PA2069	PA2069	-2.17	3.06E-03		
PA3478	*rhlB*	-2.17	1.88E-05	rhamnosyltransferase chain B	Biofilm formation
PA0535	PA0535	-2.18	3.32E-02		
PA1946	*rbsB*	-2.19	3.38E-04	binding protein component precursor of ABC ribose transporter	ABC transporters
PA1324	PA1324	-2.2	2.06E-07		
PA3788	PA3788	-2.2	7.97E-03		
PA4133	PA4133	-2.21	5.93E-05	cytochrome c oxidase subunit (cbb3-type)	Metabolic pathways
PA3906	PA3906	-2.22	3.35E-02		
PA4141	PA4141	-2.22	3.47E-07		
PA2423	PA2423	-2.23	2.55E-08		
PA0501	*bioF*	-2.23	4.30E-02	8-amino-7-oxononanoate synthase	biotin biosynthesis
PA3581	*glpF*	-2.24	4.77E-07		
PA3225	PA3225	-2.24	9.34E-06		
PA1505	*moaA2*	-2.24	4.61E-04	molybdopterin biosynthetic protein A2	Folate biosynthesis
PA5436	PA5436	-2.26	2.96E-07	probable biotin carboxylase subunit of a transcarboxylase	Biosynthesis of amino acids
PA1160	PA1160	-2.26	2.63E-02		
PA2345	PA2345	-2.26	1.14E-02	conserved hypothetical protein	Sulfur metabolism
PA1870	PA1870	-2.27	5.00E-03		
PA2176	PA2176	-2.27	6.01E-04		
PA2152	PA2152	-2.28	2.46E-03	probable trehalose synthase	Metabolic pathways
PA0526	PA0526	-2.29	1.49E-05	hypothetical protein	Denitrification
PA2164	PA2164	-2.3	5.87E-03	probable glycosyl hydrolase	Biosynthesis of secondary metabolites
PA3418	*ldh*	-2.31	7.57E-09	leucine dehydrogenase	Biosynthesis of antibiotics
PA4917	PA4917	-2.32	4.46E-03		
PA3917	*moaD*	-2.34	2.67E-02	molybdopterin converting factor, small subunit	Folate biosynthesis
PA3479	*rhlA*	-2.35	1.13E-05	rhamnosyltransferase chain A	Biofilm formation
PA3872	*narI*	-2.35	6.37E-05	respiratory nitrate reductase gamma chain	Microbial metabolism in diverse environments

PA0143	*nuh*	-2.37	1.23E-06	purine nucleosidase Nuh	Metabolic pathways
PA2300	*chiC*	-2.38	9.55E-07	chitinase	Amino sugar and nucleotide sugar metabolism
PA5506	PA5506	-2.39	1.36E-03		
PA1250	*aprI*	-2.4	4.24E-04		
PA3690	PA3690	-2.4	6.43E-11		
PA0102	PA0102	-2.41	2.28E-10	probable carbonic anhydrase	Nitrogen metabolism
PA1852	PA1852	-2.41	1.32E-05		
PA1871	*lasA*	-2.42	4.75E-07	LasA protease precursor	Quorum sensing
PA2159	PA2159	-2.43	4.78E-03		
PA4778	*cueR*	-2.43	1.33E-07		
PA2476	*dsbG*	-2.43	3.22E-02		
PA2024	PA2024	-2.45	5.38E-11	probable ring-cleaving dioxygenase	Aromatic compound catabolism
PA5528	PA5528	-2.45	4.95E-06		
PA1920	*nrdD*	-2.45	2.85E-05	class III (anaerobic) ribonucleoside-triphosphate reductase subunit, NrdD	Metabolic pathways
PA3205	PA3205	-2.45	9.43E-08		
PA4134	PA4134	-2.48	4.51E-05		
PA3192	*gltR*	-2.48	3.47E-03	two-component response regulator GltR	Glycolysis / Gluconeogenesis
PA3904	PA3904	-2.5	3.69E-03		
PA4331	PA4331	-2.5	4.20E-02		
PA2588	PA2588	-2.52	4.92E-05		
PA5498	PA5498	-2.54	1.25E-03	ZnuA	ABC transporters
PA1874	PA1874	-2.58	2.68E-09	hypothetical protein	Quorum sensing
PA0859	PA0859	-2.6	1.68E-02		
PA0523	*norC*	-2.6	2.13E-05	nitric-oxide reductase subunit C	Denitrification
PA1176	*napF*	-2.61	6.33E-03	ferredoxin protein NapF	Nitrogen metabolism
PA2964	*pabC*	-2.62	1.34E-02	4-amino-4-deoxychorismate lyase	4-aminobenzoate biosynthesis
PA1332	PA1332	-2.62	1.83E-02		
PA1732	PA1732	-2.64	1.94E-05		
PA3918	*moaC*	-2.66	2.79E-05	molybdopterin biosynthetic protein C	Folate biosynthesis
PA2665	PA2665	-2.67	2.99E-05		

PA3416	PA3416	-2.67	1.05E-12	probable pyruvate dehydrogenase E1 component, beta chain	anaerobic respiration
PA4117	*bphP*	-2.67	9.79E-11	bacterial phytochrome, BphP	Two-component System
PA3191	PA3191	-2.69	5.34E-03	glucose transport sensor, GtrS	Two-component System
PA4877	PA4877	-2.7	1.06E-04		
PA3914	*moaA1*	-2.71	1.05E-04	molybdenum cofactor biosynthetic protein A1	Folate biosynthesis
PA3724	*lasB*	-2.72	1.62E-10	elastase LasB	Cationic antimicrobial peptide (CAMP) resistance
PA2162	PA2162	-2.74	2.38E-03	probable glycosyl hydrolase	Biosynthesis of secondary metabolites
PA1227	PA1227	-2.76	4.87E-02		
PA0878	PA0878	-2.78	2.16E-03	hypothetical protein	C5-Branched dibasic acid metabolism
PA2261	PA2261	-2.79	5.62E-03	probable 2-ketogluconate kinase	glucose degradation (oxidative)
PA3045	PA3045	-2.8	4.14E-02	Two-component response regulator, RocA2	Two-component System
PA0051	*phzH*	-2.81	2.56E-05	potential phenazine-modifying enzyme	Alanine, aspartate and glutamate metabolism
PA2173	PA2173	-2.81	1.64E-02		
PA4955	PA4955	-2.83	2.77E-06		
PA2252	PA2252	-2.83	9.21E-04		
PA1429	PA1429	-2.86	8.25E-10		
PA3415	PA3415	-2.88	2.53E-08	probable dihydrolipoamide acetyltransferase	Biosynthesis of antibiotics
PA3417	PA3417	-2.93	6.38E-12	probable pyruvate dehydrogenase E1 component, alpha subunit	anaerobic respiration
PA3871	PA3871	-2.93	1.39E-08		
PA3677	PA3677	-2.95	2.80E-04		
PA2746a	PA2746a	-2.95	1.16E-05		
PA2160	PA2160	-2.96	1.04E-06	probable glycosyl hydrolase	Ascorbate and aldarate metabolism
PA1247	*aprE*	-2.97	2.77E-06	alkaline protease secretion protein AprE	Apr type I secretion system
PA3969	PA3969	-3	1.89E-04		
PA3873	*narJ*	-3.02	1.22E-06	respiratory nitrate reductase delta chain	Two-component system
PA3393	*nosD*	-3.02	1.28E-07		
PA3520	PA3520	-3.06	6.49E-05		
PA1333	PA1333	-3.06	1.80E-09		

PA2655	PA2655	-3.07	3.19E-04		
PA2116	PA2116	-3.08	1.94E-07		
PA3535	PA3535	-3.1	1.22E-04		
PA4610	PA4610	-3.12	3.30E-08		
PA2163	PA2163	-3.16	3.73E-03	hypothetical protein	Metabolic pathways
PA2830	*htpX*	-3.24	4.80E-12		
PA3928	PA3928	-3.26	1.32E-03		
PA2604	PA2604	-3.35	8.96E-24		
PA3915	*moaB1*	-3.36	2.15E-08	molybdopterin biosynthetic protein B1	molybdopterin biosynthesis
PA3396	*nosL*	-3.38	2.14E-04		
PA0918	PA0918	-3.39	2.12E-16		
PA2320	*gntR*	-3.43	1.49E-06		
PA2161	PA2161	-3.46	7.87E-03		
PA2253	*ansA*	-3.46	1.73E-03	L-asparaginase I	Alanine and Aspartate metabolism
PA0525	PA0525	-3.51	6.77E-10	probable dinitrification protein NorD	Denitrification
PA3391	*nosR*	-3.69	2.06E-11		
PA0103	PA0103	-3.78	1.65E-04		
PA2158	PA2158	-3.8	7.92E-05	probable alcohol dehydrogenase (Zn-dependent)	heterolactic fermentation
PA4364	PA4364	-3.86	5.21E-03		
PA2323	PA2323	-3.93	6.62E-22	probable glyceraldehyde-3-phosphate dehydrogenase	aerobic glycerol degradation I
PA4365	PA4365	-4.02	4.19E-03		
PA3712	PA3712	-4.06	1.87E-15		
PA4116	*bphO*	-4.16	2.04E-14	heme oxygenase, BphO	Porphyrin and chlorophyll metabolism
PA0446	PA0446	-4.17	6.59E-17		
PA1248	*aprF*	-4.21	2.50E-07	Alkaline protease secretion outer membrane protein AprF precursor	Apr type I secretion system
PA0452	PA0452	-4.32	6.80E-26		
PA2128	*cupA1*	-4.38	7.48E-08		
PA3575	PA3575	-4.46	1.05E-04		
PA2321	PA2321	-4.48	1.10E-06	gluconokinase	Biosynthesis of antibiotics
PA3432	PA3432	-4.52	5.02E-12		

PA0052	PA0052	-4.52	1.02E-13		
PA3394	*nosF*	-4.54	1.83E-06	NosF protein	ABC transporters
PA3307	PA3307	-4.59	9.58E-08		
PA1245	PA1245	-4.6	2.47E-16	AprX	Apr type I secretion system
PA3562	*fruI*	-4.68	8.95E-12	phosphotransferase system transporter enzyme I, FruI	Fructose and mannose metabolism
PA4175	*piv*	-4.82	7.31E-27	protease IV	Pyoverdine synthesis
PA3193	*glk*	-4.85	2.53E-08	glucokinase	Amino sugar and nucleotide sugar metabolism
PA3561	*fruK*	-4.86	1.75E-09	1-phosphofructokinase	Fructose and mannose metabolism
PA3194	*edd*	-4.9	3.04E-17	phosphogluconate dehydratase	Carbon metabolism
PA3181	PA3181	-4.94	2.95E-09	2-keto-3-deoxy-6-phosphogluconate aldolase	Carbon metabolism
PA0144	PA0144	-4.99	1.02E-06		
PA1246	*aprD*	-5.04	5.18E-11	alkaline protease secretion protein AprD	ABC transporters
PA3395	*nosY*	-5.18	3.38E-06	NosY protein	ABC transporters
PA3560	*fruA*	-5.23	7.40E-14	phosphotransferase system transporter fructose-specific IIBC component, FruA	Amino sugar and nucleotide sugar metabolism
PA2322	PA2322	-5.32	2.64E-12		
PA0451	PA0451	-5.41	4.61E-16		
PA1249	*aprA*	-5.52	7.11E-44	alkaline metalloproteinase precursor	Apr type I secretion system
PA0534	PA0534	-5.69	9.63E-21	FAD-dependent oxidoreductase	Polyamine catabolism
PA4714	PA4714	-5.76	2.83E-23		
PA3195	*gapA*	-6.04	1.93E-17	glyceraldehyde 3-phosphate dehydrogenase	aerobic glycerol degradation I
PA3183	*zwf*	-6.07	2.25E-17	glucose-6-phosphate 1-dehydrogenase	Biosynthesis of antibiotics
PA3914	*moeA1*	-6.21	2.44E-15	molybdenum cofactor biosynthetic protein A1	Folate biosynthesis
PA0447	*gcdH*	-6.5	9.45E-40	glutaryl-CoA dehydrogenase	Benzoate degradation
PA3431	PA3431	-7.02	1.15E-17		
PA3186	*oprB*	-8.06	4.40E-16		
PA3182	*pgl*	-8.35	1.37E-08	6-phosphogluconolactonase	Amino sugar and nucleotide sugar metabolism
PA3189	PA3189	-9.92	2.36E-10	probable permease of ABC sugar transporter	ABC transporters
PA3188	PA3188	-11.6	4.69E-10	probable permease of ABC sugar transporter	ABC transporters
PA3187	PA3187	-14.32	1.09E-17	probable ATP-binding component of ABC transporter	ABC transporters

PA3190	PA3190	-30.04	1.81E-56	probable binding protein component of ABC sugar transporter	ABC transporters

Supplementary Table 4. Primers used in this study.

Names	Sequences
rhlR-F	TCACGAGAAGTACGGGATTC
rhlR-R	TCTCGCCCTTGACCTTCTG
rhlR-1	agctcggtacccgggAAGTTCAACGTGCCCGAGCA
rhlR-2	TGCGCTTCAGATGAGACCCAGTGCAGTAAGCCCTGATCGAT
rhlR-3	ATCGATCAGGGCTTACTGCACTGGGTCTCATCTGAAGCGCA
rhlR-4	cgacggccagtgccaTTGCAGGCTGGACCAGAATA
lasR-F	GCATATCTACGATTCGGTGC
lasR-R	TACTGCGGGAAATGGGTTTG
lasR-1	agctcggtacccgggGTGATGCTGCAACTGCTCTA
lasR-2	GAGGCAAGATCAGAGAGTAACAACCAAGGCCATAGCGCTA
lasR-3	TAGCGCTATGGCCTTGGTTGTTACTCTCTGATCTTGCCTC
lasR-4	cgacggccagtgccaACAGGTCCCCGTCATGAAAC
mvfR-F	GGTGGCTACGCTAACAAAAG
mvfR-R	ACATGATGCTGGTCGACCTG
mvfR-1	agctcggtacccgggTTTCGGTCGTTAGACCTACG
mvfR-2	GCGAGCGCCTTCGGGCCTGACATCCCTTATTCCTTTTATT
mvfR-3	AATAAAAGGAATAAGGGATGTCAGGCCCGAAGGCGCTCGC
mvfR-4	cgacggccagtgccaATGCTCAAGGTCGATCGCTA
clpV1-F	GGACTATGACTTCTTCCAGG
clpV1-R	CTGGAAGTCGTAGTCGTTGA
clpV1-1	agctcggtacccgggTTCATGGACGTCTTCCACCA
clpV1-2	TCGTAGTGGAACTGGTTGTCCAGCTTGCAGAATACCGTGG
clpV1-3	CCACGGTATTCTGCAAGCTGGACAACCAGTTCCACTACGA
clpV1-4	cgacggccagtgccaCAGTAGTAGATGCCTTCCTG
clpV2-F	TCAACCTGATCGGCTTGTTC
clpV2-R	TCCAGCAGGTTTTCCGGAAA
clpV2-1	agctcggtacccgggGAACTTCCTCGATCTCTTCC
clpV2-2	CATCGCTTCCAGCAAACGGTGTTGTTGCAGGTCTACGTTG
clpV2-3	CAACGTAGACCTGCAACAACACCGTTTGCTGGAAGCGATG
clpV2-4	cgacggccagtgccaAACACGGTATAGGGGTTGTG
clpV3-F	CCCTATGCCTACCAGGAATG
clpV3-R	CTCTCTGTCTTGCTTCCGAA
clpV3-1	agctcggtacccgggTTCCTCGACCTGTTCCAGCA
clpV3-2	CCTCAAATAGGGTCCTACTCGAGTTCCATTTCAGGCTACC
clpV3-3	GGTAGCCTGAAATGGAACTCGAGTAGGACCCTATTTGAGG
clpV3-4	cgacggccagtgccaAGAGTACCCCGTCTTTATGG
clpV1-up	GGggatccCACGAGCACAAGGAGAAGGA
clpV1-down	CCaagcttTTCCTTCTACTGCTCTGCGT

Supplementary Table 5. Strains and plasmids used in this study.

Strains or Plasmids	Decription	Source
Pseudomonas aeruginosa PAO1	Wild type strain	Laboratory store
PAO1-GFP	Wild type strain with mono copy of GFP inserted into genome	This study
Δ*clpV1*-GFP	ClpV1 deletion mutant with mono copy of GFP inserted into genome	This study
Δ*clpV2*-GFP	ClpV2 deletion mutant with mono copy of GFP inserted into genome	This study
Δ*clpV3*-GFP	ClpV3 deletion mutant with mono copy of GFP inserted into genome	This study
Δ*clpV1(clpV1)-GFP*	ClpV1 complymentary strain with mono copy of GFP inserted into genome	This study
Δ*pslBCD*-GFP	PslB, PslC and PslD triple deletion mutant with mono copy of GFP inserted into genome	This study
Δ*pelA*-GFP	PelA deletion mutant with mono copy of GFP inserted into genome	This study
Δ*pilA*-GFP	PilA deletion mutant with mono copy of GFP inserted into genome	This study
Δ*fliM*-GFP	FliM deletion mutant with mono copy of GFP inserted into genome	This study
Δ*rhlR*-GFP	RhlR deletion mutant with mono copy of GFP inserted into genome	This study
Δ*lasR*-GFP	LasR deletion mutant with mono copy of GFP inserted into genome	This study
Δ*mvfR*-GFP	MvfR deletion mutant with mono copy of GFP inserted into genome	This study
Δ*rhlRlasRmvfR*-GFP	RhlR, LasR and MvfR triple deletion mutant with mono copy of GFP inserted into genome	This study
pUCP22-*clpV1*	high copy number plasmid pUCP22 with ClpV1 encording gene in E. coli DH5α, Amp^R	This study
pminiCTX-Tn7-GFP	Tn7 transposon plasmid with GFP tag in *E. coli* DH5α, Gm^R	Laboratory store
pBF13	helper plasmid in *E. coli* DH5α, Amp^R	Laboratory store
pRK600	helper plasmid in *E. coli* HB101, Cm^R	Laboratory store
pK18-Gm-mob*sacB*	suicide knock out plasmid, Gm^R	Professor Zhang Lian-Hui
